# Large language models surpass human experts in predicting neuroscience results

**DOI:** 10.1038/s41562-024-02046-9

**Published:** 2024-11-27

**Authors:** Xiaoliang Luo, Akilles Rechardt, Guangzhi Sun, Kevin K. Nejad, Felipe Yáñez, Bati Yilmaz, Kangjoo Lee, Alexandra O. Cohen, Valentina Borghesani, Anton Pashkov, Daniele Marinazzo, Jonathan Nicholas, Alessandro Salatiello, Ilia Sucholutsky, Pasquale Minervini, Sepehr Razavi, Roberta Rocca, Elkhan Yusifov, Tereza Okalova, Nianlong Gu, Martin Ferianc, Mikail Khona, Kaustubh R. Patil, Pui-Shee Lee, Rui Mata, Nicholas E. Myers, Jennifer K. Bizley, Sebastian Musslick, Isil Poyraz Bilgin, Guiomar Niso, Justin M. Ales, Michael Gaebler, N. Apurva Ratan Murty, Leyla Loued-Khenissi, Anna Behler, Chloe M. Hall, Jessica Dafflon, Sherry Dongqi Bao, Bradley C. Love

**Affiliations:** 1 Department of Experimental Psychology, University College London, London, UK; 2 Department of Engineering, University of Cambridge, Cambridge, UK; 3 Department of Physiology, Anatomy and Genetics, University of Oxford, Oxford, UK; 4 Department of Computer Science, University of Bristol, Bristol, UK; 5 Max Planck Institute for Neurobiology of Behavior – caesar, Bonn, Germany; 6 National Magnetic Resonance Research Center (UMRAM), Bilkent University, Ankara, Turkey; 7 Department of Psychiatry, Yale University School of Medicine, New Haven, CT USA; 8 Department of Psychology, Emory University, Atlanta, GA USA; 9 Faculty of Psychology and Educational Sciences, Université de Genève, Geneva, Switzerland; 10 Department of Neurosurgery, Novosibirsk State Medical University, Novosibirsk, Russia; 11 Federal Center of Neurosurgery, FSBI, Novosibirsk, Russia; 12 Department of Data Collection and Processing Systems, Novosibirsk State Technical University, Novosibirsk, Russia; 13 Department of Data Analysis, Ghent University, Ghent, Belgium; 14 Department of Psychology, New York University, New York, NY USA; 15 Department of Cognitive Neurology, University of Tübingen, Tübingen, Germany; 16 Department of Computer Science, Princeton University, Princeton, NJ USA; 17 ILCC, University of Edinburgh, Edinburgh, UK; 18 Philosophy, Psychology, and Language Sciences, The University of Edinburgh, Edinburgh, UK; 19 Department of Culture, Cognition and Computation, Aarhus University, Aarhus, Denmark; 20 Department of Molecular Life Sciences, University of Zurich, Zurich, Switzerland; 21 Department of Bioengineering, University of Pennsylvania, Philadelphia, PA USA; 22 Linguistic Research Infrastructure, University of Zurich, Zurich, Switzerland; 23 Department of Electronic and Electrical Engineering, University College London, London, UK; 24 Department of Brain and Cognitive Sciences, Massachusetts Institute of Technology, Cambridge, MA USA; 25 Institute of Neuroscience and Medicine, INM-7: Brain and Behaviour, Research Centre Jülich, Jülich, Germany; 26 Medical Faculty, Institute of Systems Neuroscience, Heinrich Heine University Düsseldorf, Düsseldorf, Germany; 27 Graduate School of Systemic Neurosciences, Ludwig-Maximilians-University Munich, Planegg-Martinsried, Germany; 28 Institute of Neuronal Cell Biology, Technical University of Munich, Munich, Germany; 29 Faculty of Psychology, University of Basel, Basel, Switzerland; 30 School of Psychology, University of Nottingham, Nottingham, UK; 31 Ear Institute, University College London, London, UK; 32 Institute of Cognitive Science, University of Osnabrück, Osnabrück, Germany; 33 Department of Cognitive, Linguistic, and Psychological Sciences, Brown University, Providence, RI USA; 34 Département de psychologie, Centre de recherche de l’Institut universitaire de gériatrie de Montréal, Montreal, Quebec Canada; 35 Instituto Cajal, CSIC, Madrid, Spain; 36 School of Psychology and Neuroscience, University of St Andrews, St Andrews, UK; 37 Department of Neurology, Max Planck Institute for Human Cognitive and Brain Sciences, Leipzig, Germany; 38 Department of Psychology, Georgia Institute of Technology, Atlanta, GA USA; 39 Département des Neurosciences Cliniques, Lausanne University Hospital, Lausanne, Switzerland; 40 School of Psychological Science, The University of Newcastle, Newcastle, New South Wales Australia; 41 Institute of Physiology, University Medical Center of the Johannes Gutenberg University, Mainz, Germany; 42 Institute for Quantitative and Computational Biosciences, Johannes Gutenberg University, Mainz, Germany; 43 Data Science and Sharing Team, Functional Magnetic Resonance Imaging Facility, National Institute of Mental Health, Bethesda, MD USA; 44 Machine Learning Team, Functional Magnetic Resonance Imaging Facility, National Institute of Mental Health, Bethesda, MD USA; 45 Zurich Center for Neuroeconomics, Department of Economics, University of Zurich, Zurich, Switzerland; 46 The Alan Turing Institute, London, UK; 47 Present Address: Valence Labs, Montreal, Québec Canada

**Keywords:** Neuroscience, Scientific community

## Abstract

Scientific discoveries often hinge on synthesizing decades of research, a task that potentially outstrips human information processing capacities. Large language models (LLMs) offer a solution. LLMs trained on the vast scientific literature could potentially integrate noisy yet interrelated findings to forecast novel results better than human experts. Here, to evaluate this possibility, we created BrainBench, a forward-looking benchmark for predicting neuroscience results. We find that LLMs surpass experts in predicting experimental outcomes. BrainGPT, an LLM we tuned on the neuroscience literature, performed better yet. Like human experts, when LLMs indicated high confidence in their predictions, their responses were more likely to be correct, which presages a future where LLMs assist humans in making discoveries. Our approach is not neuroscience specific and is transferable to other knowledge-intensive endeavours.

## Abstract (web summary)

Large language models (LLMs) can synthesize vast amounts of information. Luo et al. show that LLMs—especially BrainGPT, an LLM the authors tuned on the neuroscience literature—outperform experts in predicting neuroscience results and could assist scientists in making future discoveries.

## Main

Keeping up with the exponentially increasing^1^ scientific literature is a superhuman challenge. Potentially disruptive findings go unnoticed in the deluge of articles^2^. Processing and integrating the myriad of relevant findings may already surpass humans’ abilities.

One path forward involves human scientists leveraging advanced machines. This approach could take several forms, including specialist solutions that address specific challenges, such as in protein folding^3^, drug discovery^4^ and materials science^5^. Alternatively, general models of the scientific literature could help guide human scientists’ predictions and study designs. We consider this possibility.

It is an open question whether large language models (LLMs), trained on general text and scientific articles, can predict the outcomes of experiments. If LLMs’ predictions surpassed human experts, the practice of science and the pace of discovery would radically change. We consider this question for neuroscience, which is a large and interdisciplinary field. Prediction in neuroscience should be challenging for human experts for several reasons: (1) there are often many thousands of relevant scientific articles, (2) an individual study can be noisy or unreliable and may not replicate, (3) neuroscience is a multi-level endeavour^6^, spanning behaviour and molecular mechanisms, (4) and the analysis methods are diverse and can be complex^7^, (5) as are the methods used, which include different brain imaging techniques, lesion studies, gene modification, pharmacological interventions and so forth.

Can LLMs meet these challenges? In other domains, LLMs have performed impressively. Upon its release, OpenAI’s ChatGPT^8^ captured the public’s imagination with its abilities. Most LLMs are based on the transformer architecture^9^. These models contain billions and sometimes trillions of weights^10^, which are tuned during training in a self-supervised manner to predict the next token, such as the next word in a text passage.

LLMs have displayed remarkable capabilities, including passing professional exams, reasoning (although not without limitations), translation, solving mathematics problems and even writing computer code^11,12^. By constructing a statistical model during their training to predict the next token, whether that token is a word, pixel or protein sequence^13^, and by capturing patterns in the training data, including subtle and imperfect ones, the generative LLMs can potentially generalize to novel situations and predict outcomes of future events.

**Fig. 1:**
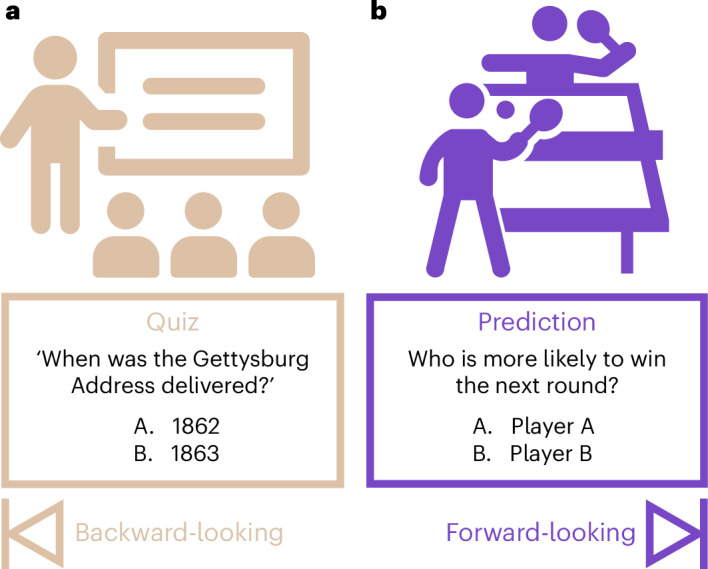
Backward-looking and forward-looking evaluations. **a**, Backward-looking benchmarks involve recalling factual information. For example, a student retrieves a fact about the Gettysburg Address that they learned during a history class. Existing benchmarks in scientific domains are in essence backward-looking as they emphasize retrieving accepted facts for question answering and reasoning tasks. **b**, Forward-looking benchmarks involve predicting novel outcomes on the basis of past data. Two forms of uncertainty, aleatoric (due to intrinsic randomness) and epistemic (due to lack of knowledge), may be present. For example, a table tennis fan predicts which player will win the next set on the basis of their knowledge of the players, how they have played so far today and so forth. Inherent random factors, such as a breeze affecting the ball’s flight, will also be present.

How can we formally evaluate the predictive abilities of LLMs in neuroscience? With the rise of LLMs, there has been a surge in evaluation benchmarks, many of which focus on assessing LLMs’ capabilities in scientific domains. Most benchmarks evaluate core knowledge retrieval and reasoning abilities, which are typically backward-looking (Fig. 1). Backward-looking benchmarks include MMLU^14^, PubMedQA^15^ and MedMCQA^16^. These benchmarks are structured in a question-and-answer format, where models must demonstrate extensive world knowledge, retrieve relevant information based on the context of the question, and answer correctly. However, none of these benchmarks is suitable for evaluating the ability of models to predict novel outcomes, which is inherently forward-looking (Fig. 1).

**Fig. 2:**
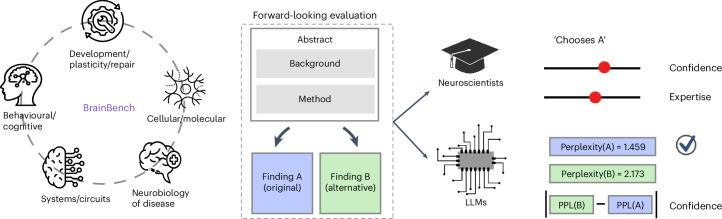
BrainBench is a forward-looking benchmark for neuroscience. BrainBench evaluates test-takers' ability to predict neuroscience results. BrainBench’s test cases were sourced from recent *Journal of Neuroscience* abstracts across five neuroscience domains: behavioural/cognitive, systems/circuits, neurobiology of disease, cellular/molecular and developmental/plasticity/repair. Test-takers chose between the original abstract and one altered to substantially change the result while maintaining coherency. Human experts and LLMs were tasked with selecting the correct (that is, original) version from the two options. Human experts made choices and provided confidence and expertise ratings in an online study. LLMs were scored as choosing the abstract with the lower perplexity (that is, the text passage that was less surprising to the model), and their confidence was proportional to the difference in perplexity between the two options.

To address this need, we developed BrainBench to test LLMs’ ability to predict neuroscience findings (Fig. 2). LLMs have been trained extensively on the scientific literature, including neuroscience. BrainBench evaluates whether LLMs have seized on the fundamental patterning of methods and results that underlie the structure of neuroscience. Can LLMs outperform human experts on this forward-looking benchmark? In particular, BrainBench evaluates how well the test-taker can predict neuroscience results from methods by presenting two versions of an abstract from a recent journal article. The test-taker’s task is to predict the study’s outcome, choosing between the original and an altered version. The altered abstract substantially changes the study’s outcome (that is, results) while maintaining overall coherence.

To appreciate how BrainBench qualitatively differs from existing benchmarks, consider a perceived limitation of LLMs, namely, their tendency to generate erroneous information, a phenomenon commonly referred to as ‘hallucination’ by LLM researchers. Unlike knowledge graphs that store verified facts, LLMs may not be trustworthy for backward-looking tasks such as summarizing research papers or providing accurate citations^17^. However, for forward-looking tasks, such as predicting results from a novel experiment, we view this tendency to mix and integrate information from large and noisy datasets as a virtue. What is a hallucination in a backward-looking task is a generalization or prediction in a forward-looking task (for example, BrainBench). BrainBench provides a way to quantify this forward-looking ability and compare with human experts. To foreshadow our results, LLMs surpassed human experts on BrainBench by a substantial margin, and this margin increased when we provided additional training in neuroscience to an LLM, which we refer to as ‘BrainGPT’.

## Results

### General-purpose LLMs best neuroscientists on BrainBench

On each benchmark trial (Fig. 2), both the LLMs^18–21^ and human experts were tasked with selecting which of two versions of an abstract was correct (that is, the original version). Human neuroscience experts were screened for their expertise and engagement (Methods) with 171 out of 202 participants passing all checks and included in our analyses.

**Fig. 3:**
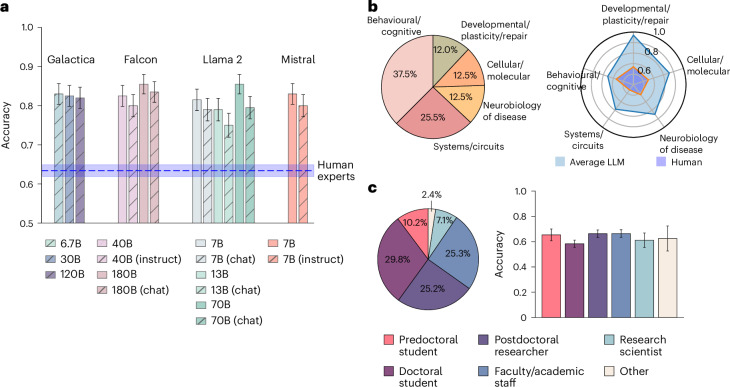
Performance of human experts and LLMs on BrainBench. **a**, LLMs outperformed human experts on BrainBench (*t*(14) = 25.8, *P* < 0.001, Cohen’s *d* = 9.27, 95% CI 0.17–0.2; two-sided). Smaller models are on par with larger models. Base versions of models outperformed chat and instruct versions (*t*(5) = 5.38, *P* = 0.002, Cohen’s *d* = 0.77, 95% CI 0.02–0.04; two-sided), which were tuned to be conversational with humans. The error bars represent the standard error of the accuracy. Each model was evaluated on 200 BrainBench test cases. In total, 171 human experts were evaluated on the same test cases over 1,011 trials. **b**, The distribution of test cases across neuroscience subfields roughly mirrors the distribution of articles in the *Journal of Neuroscience* with behaviour/cognitive overrepresented. The average performance of 15 LLMs and human experts is shown. LLMs outperformed human experts in every subfield (see Supplemetary Fig. 5 for the full results). **c**, The participants were predoctoral students (*n*_trial_ = 104), doctoral students (*n*_trial_ = 300), postdoctoral researchers (*n*_trial_ = 255), faculty/academic staff (*n*_trial_ = 256), research scientists (*n*_trial_ = 72) and others (*n*_trial_ = 24). The error bars represent the standard error of the accuracy.

Every LLM outperformed human experts on BrainBench with LLMs averaging 81.4% accuracy and human experts averaging 63.4% (*t*(14) = 25.8, *P* < 0.001, Cohen’s *d* = 9.27, 95% confidence interval (CI) 0.17–0.2; two-sided; Fig. 3a). When restricting human responses to those in the top 20% of self-reported expertise for that test item, accuracy rose to 66.2%, still below the level of LLMs.

Smaller models such as Llama2-7B and Mistral-7B with 7 billion parameters performed comparably to larger models (Fig. 3a) while besting even smaller models (Supplementary Fig. 2) that may lack the capacity to capture key data patterns. Chat or instruction-optimized models performed worse than their base model counterparts (*t*(5) = 5.38, *P* = 0.002, Cohen’s *d* = 0.77, 95% CI 0.02–0.04; two-sided). We suspect that aligning LLMs to engage in natural language conversations hinders their scientific inference abilities (Discussion).

The previous analyses involved benchmark items created by co-authors who are neuroscience experts (Methods). We conducted the same analyses using test cases generated by a LLM, namely, GPT-4 (Methods), and observed similar results (Supplementary Figs. 20, 21 and 23).

#### Performance breakdown by subfield and by participant type

BrainBench encompasses test cases from five distinct neuroscience domains: behavioural/cognitive, cellular/molecular, systems/circuits, neurobiology of disease and development/plasticity/repair. Some domains, particularly behavioural/cognitive, are overrepresented both in BrainBench (Fig. 3B) and in the *Journal of Neuroscience* from which we drew our test cases (Methods).

On average, LLMs performed better than human experts in every subfield (Fig. 3b), as did each individual LLM (Supplementary Fig. 5). Most human experts were doctoral students, postdoctoral researchers or faculty/academic staff (Fig. 3c). Please refer to Supplementary Information for more detailed demographic information including years of experience in neuroscience research about the human experts and distributions of self-reported expertise by subfield (Supplementary Fig. 17).

#### Do judgements from LLMs and human experts align?

We considered whether human experts and LLMs found the same benchmark items difficult. For humans, we calculated the mean accuracy for each of the 200 test cases. For LLMs, we calculated the signed differences in perplexity between incorrect and correct abstracts for each test case. Perplexity measures how surprising a text passage is to an LLM. Using these measures (Supplementary Fig. 6), the mean Spearman correlation between an LLM and human experts was 0.15 (±0.03), whereas the mean Spearman correlation between LLMs was 0.75 (±0.08).

#### LLMs can integrate information across context

To better understand the basis for the remarkable performance of LLMs (see Supplementary Fig. 3 for results), we investigated whether their performance was achieved by integrating information throughout the abstract (including the method used) or by solely relying on the local context in the results passages that differed between the original and altered abstract (Fig. 2)

We reevaluated the LLMs on individual sentences containing only the altered results passage (that is, local context only). LLMs performed much worse when restricted to this local context (Supplementary Fig. 3), which provides strong evidence that LLMs are integrating information across the abstract, including information on background and methods. LLM’s superior performance relative to human experts appears to arise from integrating information across the abstract.

In addition, we analysed whether LLMs benefitted from a general neuroscience context (similar to few-shot prompting) rather than integrating study-relevant information. We tested models using abstracts with sentences randomly swapped from within the same neuroscience subfield. Both original and altered abstracts were used to reevaluate LLMs’ performance. As shown in Supplementary Fig. 4, there was a significant performance decline with coherent versus swapped contexts, indicating that LLMs only partially benefit from accurate, domain-specific but non-study-relevant context.

#### LLM performance is not driven by data memorization

When LLMs perform well on a benchmark, one general concern is that the benchmark itself was part of the training set, allowing the LLM to memorize the correct answers. To address this concern, we used a commonly applied measure, the zlib–perplexity ratio, for evaluating whether LLMs have memorized passages^22^. This ratio gauges the difference between a data-agnostic compression rate of text and data-specific perplexity computed by an LLM (Methods). Passages that are hard to compress but have low perplexity are indicative of memorization.

We found no indication that BrainBench was memorized by LLMs (Supplementary Fig. 7). For comparison, we calculated the zlib–perplexity ratio for a passage that we suspected would be memorized by LLMs, namely, the Gettysburg Address. The Gettysburg Address should appear multiple times in an LLM’s training set, and indeed, it showed signs of memorization (Supplementary Fig. 7). Interestingly, for some LLMs, we know exactly what they were trained on (Supplementary Table 2). For these models, the distribution of zlib–perplexity ratios heavily overlapped for items that we knew were in the training set and for items, including BrainBench, that we knew were not in the training set. We suspect that the overlap may indicate that scientific articles, which are unlikely to repeat in training sets, are stored in LLMs as general patterns, similar to human schemas, supporting performance on tasks requiring generalization (for example, BrainBench). This hypothesis invites future study.

As a final check (Methods and Supplementary Fig. 8), we confirmed that LLMs do not perform better on items published earlier in 2023 (for example, January 2023 versus October 2023), which addresses the concern that early items are more likely to have a preprint or other precursor appear in the training set that affected BrainBench performance. Likewise, an LLM trained from scratch on the published neuroscience literature, in a manner that eliminated any possible overlap between training data and BrainBench, displayed superhuman performance^23^. All our checks indicated that BrainBench items were novel for the LLMs.

### LLMs and human experts are calibrated

**Fig. 4:**
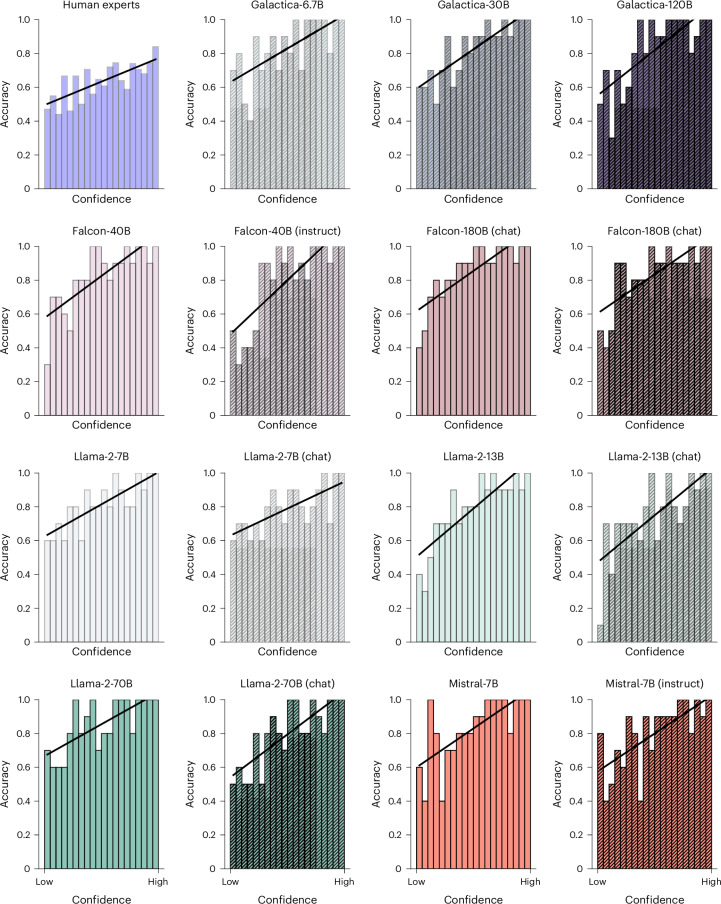
Accuracy and confidence are calibrated for human experts and LLMs. When human experts and LLMs are confident in their BrainBench judgements, they are more likely to be correct. Confidence ratings were sorted and placed in equally sized bins with the mean accuracy for items in that bin plotted. The positive slope of the black regression lines for human experts and all LLMs indicates that confidence is well calibrated (that is, higher confidence corresponds to higher accuracy). Calibration is beneficial for building human–machine ensembles.

To assess whether LLMs’ predictions are calibrated, we examined how well their confidence tracked their accuracy, a crucial characteristic for a trustworthy prediction system. We estimated LLMs’ confidence using the ranked absolute difference in perplexities between two abstracts (Fig. 2 and Methods) and found that, like human experts, all LLMs exhibited a positive correlation between accuracy and confidence. When LLMs are confident in their decisions, they are more likely to be correct (Fig. 4). In addition, we fitted logistic regressions between model perplexity differences to their correctness as well as human confidences to their correctness on the individual level. We observed significant positive correlations, confirming both models and humans are calibrated (Supplementary Table 3).

### Augmenting LLMs with neuroscience knowledge to create BrainGPT

Pre-trained LLMs can provide a foundation for further training in neuroscience with the aim of improving performance, as assessed by BrainBench. We used low-rank adaptation (LoRA)^24^ to augment a pre-trained LLM, Mistral-7B-v0.1, with additional neuroscience knowledge.

**Fig. 5:**
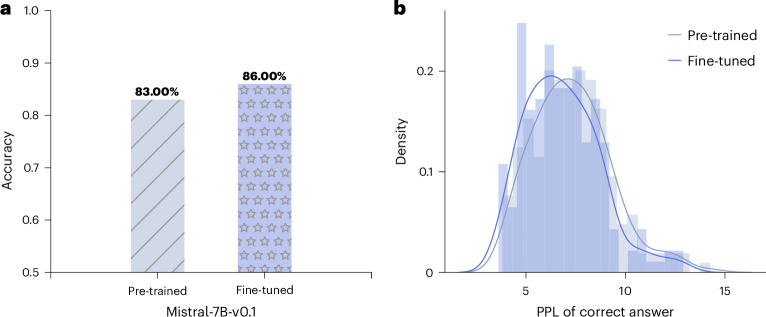
Fine-tuning a pre-trained LLM on neuroscience knowledge. Mistral-7B-v0.1 was fine-tuned using LoRA on neuroscience articles from 2002 to 2022 (a total of 1.3 billion tokens). **a**, The fine-tuned model improved by 3% on BrainBench. **b**, The fine-tuning process substantially shifted the perplexity distribution of correct responses, indicative of the LLM specializing in neuroscience.

LoRA is a parameter-efficient fine-tuning technique that inserts low-rank adapter matrices into LLM transformer blocks (Supplementary Fig. 19) and trains only these LoRA weights to update the model’s behaviour. In our case, we fine-tuned Mistral-7B-v0.1 using over 1.3 billion tokens from neuroscience publications spanning 100 journals between 2002 and 2022 (Methods), which significantly improved performance by 3% on BrainBench (Fig. 5a).

LoRA tuning dramatically shifted (*t*(199) = 15.7, *P* < 0.001, Cohen’s *d* = 0.25, 95% CI 0.42–0.55; two-sided) the perplexity of correct responses (Fig. 5b), which is indicative of the LLM specializing for neuroscience material. LoRA introduced 629,145,600 new weights, which is 8% of the total number of weights in Mistral-7B-v0.1. These results indicate that BrainGPT models can efficiently be derived by extending existing LLMs.

## Discussion

We considered whether LLMs can forecast the outcome of neuroscience experiments. By training on the vast scientific literature, we hoped LLMs could build a generative model that captured the patterns underlying neuroscience. To evaluate this possibility, we constructed a new forward-looking (Fig. 2) benchmark, BrainBench.

BrainBench assesses a test-taker’s ability to select which of two versions of a neuroscience abstract contains the actual results of the study (Fig. 2). We found that LLMs outperform human experts on BrainBench by a considerable margin (Fig. 3a) across all neuroscience subfields we considered (Fig. 3b). Moreover, when LLMs indicated high confidence in their predictions, they were more likely to be correct (Fig. 4). LLMs’ superior performance arose from their ability to integrate information throughout the abstract, such as text pertaining to the method and study design. When access to such information was removed, LLM performance drastically declined (Supplementary Fig. 3).

We found no indication that LLMs had been exposed to and memorized BrainBench items during their training. Instead, our analyses suggested that LLMs discovered the fundamental patterns that underlie neuroscience studies, which enabled LLMs to predict the outcomes of studies that were novel to them. These conclusions were supported by a widely employed technique^22^ to determine text membership within an LLMs’ training set (Supplementary Fig. 7). The Galactica^18^ LLMs were particularly illuminating because we know which articles were not in the training set versus ones that might be. Interestingly, there was no indication of memorization in models such as Galactica for scientific articles that were in its training set, consistent with the notion that LLMs learn broad patterns underlying scientific fields. While passages that frequently repeat in the training set, such as the Gettysburg Address, may be memorized (Supplementary Fig. 7), scientific articles that occur infrequently (most likely once) in the training set appear to support LLM’s forward-looking predictive abilities. As a final check, we trained a relatively small LLM from scratch^23^ on the published neuroscience literature (excluding preprints and BrainBench items), which eliminated any possible overlap between training data and BrainBench, and found superhuman performance on BrainBench (Supplementary Fig. 2).

LLM’s impressive forward-looking capabilities suggest a future in which LLMs help scientists make discoveries. To be effective, LLMs need to be kept up to date with the rapidly expanding literature. We found that LLMs could efficiently be augmented with neuroscience knowledge using LoRA^24^, boosting performance on BrainBench (Fig. 5). LoRA provides a way to create BrainGPT models by reorienting general-purpose LLMs for use in neuroscience. One can easily imagine a future in which BrainGPT is near continuously updated with new knowledge using LoRA, along with complementary approaches such as retrieval-augmented generation^17^. Retrieval-augmented generation could be used to query a database of relevant and up-to-date scientific articles for the task at hand.

In addition to keeping LLMs up to date, benchmarks should routinely be refreshed and expanded to address current needs. One challenge is that creating forward-looking benchmarks, such as BrainBench, is labour intensive and requires human expertise. To address this potential bottleneck, we created and evaluated 100 test cases using GPT-4 through a largely automated process (Methods). Although there is room for improvement, these items were close in quality to the human-created ones with 8 of the 100 items being word-for-word matches with the human-created versions. These efforts should pave the way for the rapid creation of other forward-looking benchmarks in neuroscience, as well as benchmarks for other knowledge-intensive fields. We believe high-quality forward-looking benchmarks will be critical to developing LLMs as tools for scientific discovery.

For LLMs to serve as trustworthy and effective tools or to form ensembles with humans^25^, LLMs’ outputs should include indicators of the certainty or confidence levels associated with their predictions. Fortunately, we found that LLMs’ confidence is well calibrated. When LLMs were confident in their predictions, they were more likely to be correct (Fig. 4). A second ingredient for effective teams is being diverse or complementary. LLMs have potential here as well, as the items they found difficult did not highly correlate with those human experts found difficult (Supplementary Fig. 6). These two ingredients, being well calibrated and complementary, allow systems that combine human and machine judgements to outperform either alone^26^,which holds for BrainBench^27^.

All our results, including those for calibrated confidence, were possible only because we had access to LLM weights to calculate the perplexity of passages (Fig. 2). Our approach diverged from the popular approach of prompting models for responses through natural language (that is, chat). Prompting in natural language may yield less reliable judgements and degrade model competency compared with using model probability scores or training separate classifiers directly from internal representations^28–31^. These observations underscore the importance of working with models that are as open as possible, ideally making both the weights and training set publicly available. Accordingly, we make BrainGPT available on the Huggingface platform (https://huggingface.co/BrainGPT).

Beyond serving as a tool for neuroscientists, BrainGPT can help reveal the structure of the field. In particular, we can vary BrainGPT’s training set and observe the effect on BrainBench. For example, what is the effect of including training data from related fields such as psychology? In terms of supporting prediction, we can quantify how interrelated fields are. Does it help to weight articles in the training set by their recency, citations or impact factor? In addition to these training manipulations, we can vary how testing is conducted. For example, would step-by-step thinking via chain-of-thought reasoning^32^ benefit BrainGPT? If prediction in neuroscience is akin to a deductive reasoning process, then it should. If instead, as we suspect, prediction in neuroscience is a function of many noisy intertwined signals across subfields, then chain-of-thought reasoning will not help. BrainGPT and BrainBench can help answer these meta-science questions.

We foresee a future in which LLMs serve as forward-looking generative models of the scientific literature. LLMs can be part of larger systems that assist researchers in determining the best experiment to conduct next. One key step towards achieving this vision is demonstrating that LLMs can identify likely results. For this reason, BrainBench involved a binary choice between two possible results. LLMs excelled at this task, which brings us closer to systems that are practically useful. In the future, rather than simply selecting the most likely result for a study, LLMs can generate a set of possible results and judge how likely each is. Scientists may interactively use these future systems to guide the design of their experiments.

One risk is that scientists do not pursue studies when their predictions run counter to those of an LLM. In some cases, this might be a sensible course of action, whereas in other cases the LLM might have identified potential gaps or errors in the scientific literature. In the latter situation, conducting the study might result in a significant breakthrough. Conversely, a study result that was predicted with high confidence by an LLM might be viewed as an incremental advance.

LLMs’ predictions are informed by a vast scientific literature that no human could read in their lifetime. As LLMs improve, so should their ability to provide accurate predictions. In this contribution, we focused on neuroscience but our aims are broader; we hope to provide a template for any knowledge-intensive field. None of the approaches we adopted is neuroscience specific. Indeed, the degree of efficacy of our approach may depend on the underlying structure of the domain. For instance, disciplines like mathematics, which rely heavily on logical deduction, might not benefit as much as other scientific fields that involve pattern-based reasoning.

We hope to democratize the use of LLMs in science and increase reproducibility by highlighting the use of relatively small models that can be run locally and whose weights are accessible, which contrasts with commercial products. Finally, while LLMs appear poised to supplant humans at prediction, we foresee a role for human experts in providing the accompanying scientific explanations. Prediction is very important, but not everything.

## Methods

We confirm that our research complies with all relevant ethical regulations. Experimental Psychology Ethics Board (University College London) approved the study protocol (ethics protocol EP/2017/011). We confirm that informed consent was obtained from all human participants. Participant compensation is not applicable to the current study. None of our studies was pre-registered.

### Dataset creation

Co-authors (Supplementary Table 5) and GPT-4 (Azure OpenAI API; version 2023-05-15) created test cases that formed BrainBench. All test cases were sourced from *Journal of Neuroscience* abstracts published in 2023 under the Creative Commons Attribution 4.0 International License (CC-BY). The abstracts are organized into five sections, namely, behavioural/cognitive, systems/circuits, neurobiology of disease, development/plasticity/repair and cellular/molecular. In constructing BrainBench, we incorporated a total of 200 test cases crafted by human experts and an additional 100 test cases generated by GPT-4 (Azure OpenAI API; version 2023-05-15). All test cases were subjected to extensive quality control by human experts and GPT-4. For the distribution of test cases among subfields, refer to Fig. 3 for human-created cases and Supplementary Fig. 23 for GPT-4 generated cases.

To create a test case, a published abstract was modified to create an altered version. The altered version substantially changed the results without changing the methods and background. Minimal changes were made that changed the basic result. For example, the altered abstract, compared with the original, could switch around the role of two brain regions in the results, reverse the direction of a result (for example, replace ‘decreases’ with ‘increases’) and so on. Any changes maintained the coherency of the abstract, which sometimes required multiple changes (for example, replacing multiple ‘decreases’ with ‘increases’). In other words, the altered abstracts needed to be empirically different but not logically incoherent. Both volunteers and GPT-4 are given instructions that follow the essential criteria above. The exact wordings to prompt GPT-4 were slightly adjusted to obtain good-quality test cases. We include the instructions given to GPT-4 verbatim below.

#### GPT-4 test creation prompt

‘Your task is to modify an abstract from a neuroscience research paper such that the changes significantly alter the result of the study without changing the methods and background. This way we can test the Artificial Intelligence understanding of the abstract’s subject area.

Please read the instructions below and ensure you follow them one by one while you are modifying the abstracts:

- The format to submit is putting double brackets around the change with the first element being the original and the second element being your edit. E.g., [[original passage, modified passage]]. Always remember to wrap your edits with the double brackets; there should not be any other edits outside the brackets to the original abstract. - If you change a single word, never wrap the entire sentence inside the double brackets. For example, ‘… exhibit [[enhanced LTP and deficits in LTD, impaired LTP and enhanced LTD]].’ is a wrong format, the correct format is: ‘… exhibit [[enhanced, impaired]] LTP and [[deficits, enhanced]] in LTD.’ - The beginning of an abstract is the background and methods, so you should not alter those parts of the abstract. Do not alter the first couple sentences. - We want the abstract to become empirically wrong, but not logically incoherent. - To find the original result of the paper, one should require some neuroscience insight, not just general reasoning ability. So it is critical that the changes you make don’t evaluate the Artificial Intelligence reasoning ability, but its knowledge of neuroscience and how the brain works. - Watch out for making changes that alter the results, but may still have occurred in the authors’ study. For example, an fMRI abstract on learning might mention the hippocampus and not the striatum. Nevertheless, the striatum might have also been active and not reported in the abstract because it was not the focus of the study. - The changes you make should not be identifiable or decodable from the rest of the abstract. Hence, if you make a change, make sure you change everything that can reveal the original abstract. For example, ‘activation of neurons in the visual cortex [[increases, decreases]] the activity in the motor cortex. This decrease in the activity of the visual cortex was followed by an increase in task performance.’. In this case it is very clear that the correct word is ‘decreases’ as the next sentence (‘This decrease in the activity of the visual cortex’) reveals that. - Be mindful of the article when you change words. For example, if you change the word ‘decline’ to ‘enhancement’, you must change the article as well, so the change will be [[a decline, an enhancement]]. - Ensure that your edits maintain inter-sentence consistency and proper syntax. The changes should not contradict or confuse the overall meaning of the abstract. - Avoid making trivial edits that do not require understanding of scientific concepts. The edits should reflect a deep understanding of the subject matter. - Do not miss any crucial results or findings in the abstract while making the edits. Every significant point should be addressed in your modifications.

To generate better responses, you can use the topic of their study and purpose of studies in those topics. This knowledge helps you to find what modification you should do in the abstract. Topics are: - Behavioral/Cognitive: To understand how the brain influences behavior, cognition, and emotion, and to apply this understanding in diagnosing and treating neurological and psychiatric disorders.

- Cellular/Molecular: To study are to understand the functions and mechanisms of neurons at a cellular and molecular level, which includes investigating the biology of nerve cells, their genetic makeup, and how they form complex circuits, ultimately contributing to our understanding of brain function, behavior, and the development of treatments for neurological disorders.

- Neurobiology of Disease: To understand the biological basis of various neurological and psychiatric disorders in order to develop effective treatments and preventative measures.

- Development/Plasticity/Repair: to understand the mechanisms of brain development, adaptation, and repair in response to injury or disease, with the goal of developing strategies and treatments to enhance brain recovery and function.

- Systems/Circuits: to understand how neural circuits in the brain interact and coordinate to process information, control behavior, and support cognitive functions.

Here are two examples of the edited abstract by human experts which can help you to understand the task:

Example 1: < example_1 >

Example 2: < example_2 >

These are some common mistakes you have made in the past. So keep them in mind whilst generating your responses: - You misunderstood/ignore the information provided at the beginning of the abstract. - The edits you have made are not what we are aiming for, you tweaked a portion of the studies with non-significant findings, so there’s no significant alternation of results occurring. Make sure your edit changes the main results of the studies, not trivial changes. - Lack of inter-sentence consistency in the prompt - You made edits as early as the first sentence. THe first few sentence are general knowledge and are not result of the study. So you shouldn’t make any change in the beginning. - Most of your edits contradict the conclusion. Make sure your changes do not contradit the conclusions or any part of the abstract. - You only modified verbs the understanding of which does not require understanding of scientific concepts & names of compounds, which makes the edits less likely to do wrong as long as reasons logically - One of your edits contradicts all other edits. - Your edit is inconsistent with the beginning of the sentence - You failed to change the first part of the conclusion for consistency - You missed out on one change. - You misunderstood the purpose of the study. Although in the abstract it explicitly states the purpose of the study.

Below, you are given an abstract with its topic. Follow the instructions given to you and return the modified abstract. Remember to use double brackets to show the changes ([[original, modified]] and keep the rest of the abstract unchanged. Also, pay attention to all the information you were given above as well as the common mistakes you have made before.

Abstract to edit: Topic: < abstract_topic >

Abstract: < abstract_to_edit > ’

### Evaluations

We tested human participants and LLMs on the BrainBench dataset. Both human experts and models were presented with two versions of the abstract, one with the actual results and one that was altered. The task was to determine which is which. Below, we detail how LLMs and human participants were tested.

#### Model evaluation

We tested LLMs by adapting the Eleuther AI Language Model Evaluation Harness framework^31^, which evaluates LLMs using a multiple choice setting. We presented LLMs with two versions of the abstracts from each test case separately. We prefixed each abstract with the prompt ‘You are a neuroscientist with deep knowledge in neuroscience. Here is an abstract from a neuroscience publication:’ and applied model-specific instruction templates where appropriate. We then measured the perplexity of both passages and used perplexity as the indicator of whether LLMs favour one abstract or the other.

Perplexity (PPL) is one of the most common metrics for evaluating LLMs. Perplexity measures the degree of uncertainty of a model when generating a particular sequence of text. Formally, perplexity is defined as the exponentiated average negative log-likelihood of a tokenized sequence. If we have a tokenized abstract *X* = (*x*_0_, *x*_1_, …, *x*_*t*_), then the perplexity of *X*, given a LLM parameterized by *θ*, is

1 $${\mathrm{PPL}}(X)=\exp \left\{-\frac{1}{t}\mathop{\sum }\limits_{i}^{t}\log {p}_{\theta }({x}_{i}| {x}_{ < i})\right\},$$

where $\log {p}_{\theta }({x}_{i}| {x}_{ < i})$ is the log-likelihood of the *i*th token conditioned on the preceding tokens *x*_<*i*_ according to the LLM. Given both the original abstract *X*_orig_ and the altered abstract *X*_alt_, we followed the decision rule

2 $${X}_{{\mathrm{chosen}}}=\left\{\begin{array}{ll}{X}_{{\mathrm{orig}}},\quad &\,\text{if}\,\,{\mathrm{PPL}}({X}_{{\mathrm{orig}}}) < {\mathrm{PPL}}({X}_{{\mathrm{alt}}})\\ {X}_{{\mathrm{alt}}},\quad &\,\text{otherwise}\,\end{array}\right.$$

and evaluated the overall accuracy over the entire BrainBench accordingly.

##### Accuracy

Accuracy is the primary metric for reporting LLM performance on BrainBench. A correct response was when the model produces a lower perplexity for the original abstract than the altered abstract.

##### Confidence calibration

We used the absolute difference of perplexities of two versions of the abstract as a measure of model confidence. To assess the calibration of LLMs, we compared their accuracies with their confidence levels. First, we ranked and sorted model confidence across all test cases. Subsequently, we created 20 bins based on this sort. Within each bin, we calculated the mean accuracy. A well-calibrated model will exhibit a higher accuracy in bins associated with higher confidence rankings. We fit a linear regression model using the bin number as the independent variable and the mean accuracy of each bin as the dependent variable to evaluate calibration.

##### Performance correlation across LLMs

We assessed the correlation in performance among different LLMs by examining how they rank the relative difficulty of test cases. To determine difficulty, we calculated the difference in perplexity between incorrect and correct abstracts for each test case. Intuitively, a large positive difference in the perplexity between incorrect and correct versions of an abstract should indicate that the test case is easy from the LLM’s perspective. We calculated the Spearman correlation coefficient of these difficulty measures to assess the agreement between two LLMs.

##### Integration analysis

To investigate the extent to which LLMs can integrate broad context from abstracts, we conducted an experiment involving the removal of contextual information from BrainBench test cases. Following the same evaluation procedure as previously outlined for full abstract cases, we assessed the models using individual sentences extracted from abstracts containing at least one result alternation. In cases with multiple alternations, we computed the mean accuracy across these alternations as the final accuracy for the abstract. We then compared the level of performance degradation when LLMs were evaluated on full-length abstracts versus individual sentences where background and method information from the abstracts was removed.

In addition, we tested models using abstracts whose results (in terms of complete sentences) are randomly swapped from abstracts within the same neuroscience subfield. Importantly, in these ‘swapped’ abstracts, the number of results remained consistent with the original. We applied the swapping to both original and altered abstracts and reevaluated LLMs’ performance.

##### LLM training data memorization analysis

One concern regarding LLMs outperforming human experts on BrainBench is the possibility that LLMs were exposed to the original abstracts during their pre-training. If LLMs have simply memorized the training data, they would naturally assign lower perplexity scores to the correct abstracts.

To address this concern, we employed a common method from the literature to determine whether a given text is part of LLM’s training data^22,33^. This method involves calculating the zlib entropy and the perplexity ratio (equation (3)) of a text sequence to infer its membership status:

3 $${\mathrm{ratio}}=\frac{{\mathrm{ZLIB}}(X\;)}{{\mathrm{PPL}}(X\;)}.$$

Zlib entropy is computed using the zlib text compression algorithm^34^, which measures the level of uncertainty in a text when compressed. It is a data-agnostic way of evaluating text. On the other hand, LLM perplexity depends on the specific training data and, thus, is data dependent. In general, if a piece of text surprises zlib but not LLM, it is probably part of the training data.

To conduct this test, we carefully chose data sources that are either known to be part of LLMs’ pre-training or reasonably assumed to be excluded from it (refer to Supplementary Tables 1 and 2). We then applied zlib compression and LLM perplexity calculations to text samples from these selected sources.

In addition, we introduced the Gettysburg Address as a special anchor point to contrast with the zlib–perplexity ratio distribution across multiple data sources. This is because we expect the Gettysburg Address to exhibit a high zlib score due to its non-modern form of English, coupled with a low perplexity, given its probably frequent exposure during LLM pre-training.

Finally, we analysed the Spearman correlation between the publication dates of the abstracts that make up BrainBench test cases against the test cases’ difficulties to LLMs. This was to address the concern that early items are more likely to have a preprint or other precursor appear in the training set memorized by LLMs. If there was memorization, we would expect a negative correlation between publication date and difficulty. We determined difficulty by using the difference in perplexity between incorrect and correct abstracts for each test case.

#### Human evaluation

##### Participants

We recruited 202 neuroscience experts via social media and an email newsletter. We excluded 31 participants for failing to answer both catch trials correctly, not providing confidence or expertise ratings during the entire experiment, and self-reported cheating. The remaining 171 participants consisted of 51 doctoral students, 43 faculty/academic staff, 43 postdoctoral researchers, 18 predoctoral students, 12 research scientists and 4 classified as ‘other’. Participants’ mean experience in neuroscience was 10.1 years. Participants identified as follows: 62.5% male, 34.5% female and 0.6% gender variant/non-conforming. The mean age was 35.2 years (standard deviation 9.4 years).

##### Procedure

First, participants were briefed on the experimental task and provided their informed consent to proceed to the experiment. Demographic information was then collected, including gender identity, age, country, current position and years of experience in neuroscience research, broadly construed. Next, participants completed a practice trial using the same testing format as the actual test cases. This trial was used to familiarize participants with the format of the task, with the screen proceeding only once participants had made the correct choice based on common sense. Following this, nine test trials and two catch trials commenced, where participants selected one version of each trial abstract. Out of the nine test trials, six were randomly sampled human-created test cases and three were randomly sampled from the pool of machine-created items. We ensured that each test case is sampled approximately an equal number of times across all participants. To achieve this, we maintained a global counter that keeps track of how frequently each test case has been used. As a result, the next participant’s sample will always be drawn from those test cases that have been used less frequently. Notably, the number of alterations varies between test cases, but the design allowed a single click to automatically select between the two abstract options (Supplementary Fig. 1). Participants made one decision per test case, regardless of the number of alternations.

Subsequently, participants were required to rate their confidence and expertise using slider bars. The confidence slider had a range from ‘lower’ on the left to ‘higher’ on the right, while the expertise slider spanned from ‘not at all’ on the left to ‘very much so’ on the right, both internally implementing a 1–100 scaling. In addition, participants indicated whether they had encountered the study previously before proceeding to the next trial. Upon completing the 11 trials, participants were debriefed on which trials they got correct and were subsequently asked to indicate whether they engaged in any form of cheating during the study. We hosted the study entirely on the Gorilla platform^35^.

##### Exclusion criteria

For participant selection and data analysis, we apply several exclusion criteria. First, individuals who failed to answer both catch trials correctly were not included in the data analyses. Second, participants who did not make adjustments to the sliders (that is, expertise and confidence) during any of the trials were excluded. In addition, trials where participants recognized the abstract content were omitted from the analysis. Furthermore, trials with reaction times less than 5 s were excluded. Lastly, participants who admitted to using external resources or engaging in cheating behaviours, as indicated by a checkbox in the debriefing form, were not considered in the final data analysis.

##### Performance correlation between humans and LLMs

We assessed the agreement between humans and LLMs using a similar approach as we did when evaluating the correlation among LLMs. For LLMs, the procedure for determining item difficulty was identical to that described above. For human experts, item difficulty was calculated as the mean accuracy for that item. Finally, the Spearman correlation of these difficulty measures was calculated to assess agreement.

### Fine-tuning on neuroscience corpora

The LLMs we considered had been pre-training on a diverse range of text corpora, including Internet sources, Wikipedia, books, code repositories and arXiv papers. While these pre-trained models are designed to be versatile and capable of handling various tasks, our approach for creating BrainGPT involved enhancing base models with domain-specific expertise, specifically in neuroscience.

To accomplish this, we employed the LoRA technique (Supplementary Fig. 19 and ref. ^24^). LoRA efficiently extends the capabilities of general-purpose LLMs by introducing low-rank trainable parameters (referred to as ‘adapters’) into the existing model. This process effectively fine-tunes the model for downstream tasks without the need for prohibitively resource-intensive training of the entire model.

#### Training data

We collected training data from PubMed for abstracts and PubMed Central Open Access Subset (PMC OAS) for full-text articles using the Entrez Programming Utilities (E-utilities) API (application programming interface) and the pubget Python package, respectively. The data span publication dates from 2002 to 2022. For science general journals, we applied a keyword filter of ‘Neuroscience’ (see all sourced journals in Supplementary Table 4).

Our data extraction efforts yielded 332,807 abstracts and 123,085 full-text articles, totalling 1.3 billion tokens. We excluded figures and tables and randomly allocated 90% of the data for training, reserving the remaining 10% for validation.

#### Training details

We fine-tuned Mistral-7B-v0.1^21^ using weights available on Huggingface (https://huggingface.co/mistralai/Mistral-7B-v0.1). We used a batch size of 1 and a chunk size of 2,048. Training involved the use of the AdamW optimizer^36^ with a learning rate of 2 × 10^−5^ and gradient accumulation steps set at 8. Two training epochs were performed, along with a warm-up step of 0.03 and a weight decay rate of 0.001. The learning rate was controlled using a cosine learning rate scheduler. LoRA adapters, characterized by a rank of 256, an alpha value of 512 and a dropout rate of 0.1, were applied after all self-attention blocks and fully connected layers. This results in total 629,145,600 trainable parameters, roughly 8% of the entire parameters of the base model. To optimize training performance, bf16 mixed precision training and data parallelism were employed. We used four Nvidia A100 (80 GB) graphics processing units hosted on the Microsoft Azure platform. An epoch of training takes roughly 65 graphics processing unit hours.

#### Evaluation

We tested the fine-tuned model on BrainBench using the same procedure as before. To verify the significance of performance improvement, we performed a paired *t*-test with respect to the perplexity of the correct options before and after fine-tuning.

### Reporting summary

Further information on research design is available in the Nature Portfolio Reporting Summary linked to this article.

## Supplementary information

Supplementary Information Supplementary Discussion, Tables 1–5 and Figs. 1–26.

Reporting Summary

## Data Availability

Human participant data, and intermediate data generated via simulations and analyses, are publicly available via GitHub at https://github.com/braingpt-lovelab/BrainBench. Model weights and training data are available at https://huggingface.co/BrainGPT. Model training data are sourced from PubMed and PubMed Central Open Access Subset (PMC OAS) using the Entrez Programming Utilities (E-utilities) API and the pubget Python package, respectively.

## Code availability

All computer code associated with this work including model training, evaluation, data processing and analyses are publicly available via GitHub at https://github.com/braingpt-lovelab/BrainBench.
