# Supplementary material for: Large language models surpass human experts in predicting neuroscience results
Source: Nat Hum Behav. 2024 Nov 27;9(2):305–15. doi: 10.1038/s41562-024-02046-9 (PMC11860209; doi:10.1038/s41562-024-02046-9)
Supplement: Supplementary file 1 — Supplementary Discussion, Tables 1–5 and Figs. 1–26. [file 41562_2024_2046_MOESM1_ESM.pdf]

nature human behaviour

Supplementary information https://doi.org/10.1038/s41562-024-02046-9

Large language models surpass human experts in predicting neuroscience results

In the format provided by the authors and unedited

Supplementary Information

Supplementary Discussion

Relative perplexities evaluated at token and sentence levels

To gain further insights into how models solve BrainBench test cases, we broke down the change of relative perplexities over the sequence of tokens and sentences starting from the first alternation of results. Specifically, we looked at how often the model starts correctly at the first alternation and remains correct (Pos to Pos) or becomes incorrect (Pos to Neg) at the end. In addition, we categorized how often the model starts incorrectly but becomes correct (Neg to Pos) or remains incorrect (Neg to Neg).

Examples (Fig. S.9, S.10, S.11) show different patterns of relative perplexity changes between the altered and original abstracts over tokens. These examples demonstrate that the model does not always recognize the correct version at the first alternation, nor does it consistently remain in the correct (positive) or incorrect (negative) region across tokens.

To gain a holistic view, we examined all test cases and categorized how the model solves a question. Specifically, we looked at how often the model starts correctly at the first alternation and remains correct (Pos to Pos) or becomes incorrect (Pos to Neg) at the end. We also categorized how often the model starts incorrectly but becomes correct (Neg to Pos) or remains incorrect (Neg to Neg). The corresponding results are shown in Fig. S.15 (left panel). For more than half the abstracts, if the model is correct at the first alternation, it is likely to be correct at the end. Notably, 20% of abstracts classified incorrectly at the first alternation are later classified correctly. We further evaluated beyond the first and final tokens by examining the number of sign flips of perplexity difference in between all tokens. As shown in Fig. S.15 (right panel), the model exhibits fewer sign flips for abstracts eventually classified correctly than for those classified incorrectly. We performed the same analysis at the sentence level and observed

similar patterns (Fig. S.12, S.13, S.14, S.16).

On the relationship between performance, model size and training data

What factors might be driving LLMs' performance on BrainBench? As shown in Fig. 3A, models of varying sizes starting from 7 billion parameters perform similarly, with instruct/chat fine-tuned models performing worse and larger models do not necessarily achieve higher accuracy. To gain better insights, we trained a much smaller model, GPT-2 (124 million parameters) from scratch, entirely on the same neuroscience data utilized in this study. We trained dedicated tokenizer on the same data. We trained GPT-2 for 5 epochs until convergence and tested it on BrainBench. The model achieved 63% accuracy [33], which is very close to human-level performance but falling behind on bigger models. To map out the picture more clearly, we further trained GPT-2 medium (355M) and GPT-2 large (774M) following the same training procedure. They both outperformed GPT-2 (124M) with GPT-2 large achieving a 72% accuracy further reducing the gap to much larger models we tested (see full results in Fig. S.2). This pattern of result suggests that the size of the model likely plays a critical role in integrating deep knowledge to provide accurate responses.

In addition, we tested another small LLM, TinyLLama (1.1B; v1.0), as a comparison, and the model achieved 70.5% (base) and 65.5% (chat) on BrainBench; falling much behind the 7B models we tested in the original study. Further, we conducted testing on Phi-3 (3.8B), a very recent (April, 2024) model developed by Microsoft renowned for its competitive edge even against models more than twice its size. Phi-3 achieved an 82.5% accuracy on BrainBench. We attribute this performance to the known high-quality training data curated by Microsoft.

In light of all these results, we posit that achieving superhuman performance on BrainBench likely stems from a synergy between model size and training set quality. The Phi-3 result raises intriguing questions about the existence of a scaling law in this context. It prompts exploration

into whether there's a point of diminishing returns in model scaling and whether there exists a balance between model size, data quality and relevancy, and computational resources for optimal performance on BrainBench.

Supplementary Tables

Model Series	Pretraining Data Cutoff	Initial Release Date
Llama2	September 2022	July 2023
Galactica	July 2022	November 2022
Falcon	December 2022	May 2023
Mistral	Unknown	September 2023

Table S.1: Overview of LLMs Training Cutoff and Initial Release Dates.

Data Source	Model Training Set Inclusion
RefinedWeb	Known to be in Falcon's training set[34]
Arxiv (Jun-Dec 2021)	Likely to be in Llama2's training set; known to be in Galactica's training set
Arxiv (Jun-Dec 2023)	Known to not be in Llama2 and Galactica's training set[20,35]
Biorxiv (Jun-Dec 2021)	Known to be in Galactica's training set[20]
Biorxiv (Jun-Dec 2023)	Known to not be in Galactica's training set
Gettysburg Address	Likely to be in all models' training set

Table S.2: Data Source Inclusion in LLM Training Sets

Model/Human	Coefficient	Intercept	Test Statistics
Galactica-6.7B	1.69 ± 0.04	1.18 ± 0.04	$t(4) = 42.65, p = 1.81 \times 10^{-6}$
Galactica-30B	2.03 ± 0.04	1.11 ± 0.02	$t(4) = 54.55, p = 6.76 \times 10^{-7}$
Galactica-120B	2.32 ± 0.12	0.92 ± 0.08	$t(4) = 19.97, p = 3.71 \times 10^{-5}$
Falcon-40B	2.03 ± 0.08	1.09 ± 0.06	$t(4) = 24.64, p = 1.61 \times 10^{-5}$
Falcon-40B (instruct)	2.50 ± 0.09	0.83 ± 0.03	$t(4) = 28.16, p = 9.46 \times 10^{-6}$
Falcon-180B	1.96 ± 0.08	1.36 ± 0.06	$t(4) = 25.90, p = 1.32 \times 10^{-5}$
Falcon-180B (chat)	1.97 ± 0.10	1.02 ± 0.07	$t(4) = 18.99, p = 4.53 \times 10^{-5}$
Llama-2-7B	1.43 ± 0.06	1.32 ± 0.06	$t(4) = 24.53, p = 1.64 \times 10^{-5}$
Llama-2-7B (chat)	1.39 ± 0.09	0.93 ± 0.05	$t(4) = 15.82, p = 9.32 \times 10^{-5}$
Llama-2-13B	1.92 ± 0.07	1.13 ± 0.03	$t(4) = 28.74, p = 8.72 \times 10^{-6}$
Llama-2-13B (chat)	2.14 ± 0.02	0.59 ± 0.03	$t(4) = 122.38, p = 2.67 \times 10^{-8}$
Llama-2-70B	1.43 ± 0.07	1.63 ± 0.09	$t(4) = 21.27, p = 2.89 \times 10^{-5}$
Llama-2-70B (chat)	2.30 ± 0.14	0.71 ± 0.04	$t(4) = 16.32, p = 8.25 \times 10^{-5}$
Mistral-7B	1.76 ± 0.03	1.28 ± 0.03	$t(4) = 55.71, p = 6.21 \times 10^{-7}$
Mistral-7B (instruct)	1.90 ± 0.08	1.01 ± 0.06	$t(4) = 23.74, p = 1.87 \times 10^{-5}$
Human experts	0.01 ± 0.00	0.02 ± 0.02	$t(4) = 17.34, p = 6.49 \times 10^{-5}$

Table S.3: **Logistic regression fits.** For models, logistic regressions were fitted between perplexity differences of a test case and its correctness given a LLM. For human experts, logistic regressions were fitted between their confidence of a test case and its correctness. The significant positive correlations between model and human fits suggest that LLMs and humans are calibrated. Coefficients and intercepts were learned across five random folds of data. One-sample t-Test was performed to determine the significance of the coefficients. P values are two-sided.

Nature, Cell, Cell Reports, eLife, Science Advances, Nature Communications, PNAS, The EMBO Journal, Nature Neuroscience, Neuron, Brain, NeuroImage, Molecular Psychiatry, Journal of Neuroscience, Nature Reviews Neuroscience, Cerebral Cortex, Annals of Neurology, Human Brain Mapping, Epilepsia, Clinical Neurophysiology, Trends in Cognitive Sciences, Biological Psychiatry, Translational Psychiatry, Neuroscience and Biobehavioral Reviews, Neuropsychopharmacology, Alzheimer's and Dementia, NeuroImage: Clinical, Neurobiology of Aging, Trends in Neurosciences, Nature Reviews Neurology, Brain Stimulation, Frontiers in Neuroscience, Movement Disorders, Nature Human Behaviour, Frontiers in Neurology, Cortex, Journal of Alzheimer's Disease, Neurobiology of Disease, Biological Psychiatry: Cognitive Neuroscience and Neuroimaging, Brain Structure and Function, Pain, Frontiers in Human Neuroscience, eNeuro, Current Opinion in Neurobiology, European Journal of Neuroscience, Frontiers in Aging Neuroscience, Alzheimer's Research and Therapy, Journal of Neurology, Glia, Epilepsy and Behavior, Brain Imaging and Behavior, Journal of Neurophysiology, Sleep, Neuroscience, Neuropsychologia, Journal of Neural Engineering, Molecular Neurobiology, Frontiers in Cellular Neuroscience, Neuropharmacology, Alzheimer's and Dementia: Diagnosis, Assessment and Disease Monitoring, Journal of Neuroinflammation, Epilepsia Open, Acta Neuropathologica Communications, Frontiers in Neuroinformatics, Current Opinion in Behavioral Sciences, Developmental Cognitive Neuroscience, Frontiers in Molecular Neuroscience, Cerebellum, Journal of Cognitive Neuroscience, Network Neuroscience, Annual Review of Neuroscience, Progress in Neurobiology, Epilepsy Research, Molecular Autism, Journal of Comparative Neurology, Social Cognitive and Affective Neuroscience, Brain Topography, Hippocampus, Seizure: the journal of the British Epilepsy Association, Psychophysiology, Frontiers in Behavioral Neuroscience, Journal of Neurotrauma, Journal of Physiology, Frontiers in Neural Circuits, Neurobiology of Learning and Memory, Journal of Neural Transmission, Frontiers in Neuroanatomy, International Journal of Neuropsychopharmacology, Neuroscientist, Brain Sciences, Behavioural Brain Research, Experimental Neurology, Progress in Neuro-Psychopharmacology and Biological Psychiatry, Neurological Sciences, Neurotherapeutics, Neuroscience Letters, Current Opinion in Neurology, Journal of Neuroscience Methods, Journal of Neurochemistry, Neuromodulation, Molecular Neurodegeneration, Frontiers in Systems Neuroscience, Sleep Medicine Reviews, Brain and Behavior, Brain Research, Neurorehabilitation and Neural Repair, Autism Research.

Table S.4: **Journals used for LoRA fine-tuning.** Abstracts and full articles published between 2002 and 2022 sourced from the above journals were used as the training set for LoRA fine-tuning.

Tasks	Authors
Primary writing	Xiaoliang Luo, Bradley C. Love
Test case creation	Bati Yilmaz, Isil Poyraz Bilgin, Anton Pashkov<br>Tereza Okalova, Alexandra O. Cohen, Felipe Yáñez<br>Elkhan Yusifov, N Apurva Ratan Murty, Kangjoo Lee<br>Valentina Borghesani, Sepehr Razavi, Justin M. Ales<br>Rui Mata, Michael Gaebler, Guiomar Niso<br>Leyla Loued-Khenissi, Anna Behler, Kaustubh R. Patil<br>Mikail Khona, Roberta Rocca, Kevin K. Nejad<br>Alessandro Salatiello, Jonathan Nicholas<br>Daniele Marinazzo, Chloe M. Hall<br>Pui-Shee Lee, Sebastian Musslick<br>Nicholas E. Myers, Jennifer K Bizley, Sherry Dongqi Bao<br>Nianlong Gu, Jessica Dafflon, Bradley C. Love
Quality control	Bati Yilmaz, Kevin K. Nejad, Daniele Marinazzo<br>Pui-Shee Lee, Nianlong Gu, Chloe M. Hall<br>Kangjoo Lee, Sebastian Musslick, Akilles Rechardt<br>N Apurva Ratan Murty, Ilia Sucholutsky, Guiomar Niso<br>Isil Poyraz Bilgin, Rui Mata, Tereza Okalova<br>Mikail Khona, Justin M. Ales, Michael Gaebler<br>Elkhan Yusifov, Leyla Loued-Khenissi, Jessica Dafflon<br>Jonathan Nicholas, Felipe Yáñez, Roberta Rocca<br>Valentina Borghesani, Sherry Dongqi Bao<br>Alexandra O. Cohen, Alessandro Salatiello<br>Xiaoliang Luo, Bradley C. Love
GPT-4 case creation	Kevin K. Nejad, Xiaoliang Luo, Bradley C. Love
Human-machine teaming	Felipe Yáñez, Xiaoliang Luo, Bradley C. Love
LoRA fine tuning	Guangzhi Sun, Xiaoliang Luo, Bradley C. Love
Model evaluation	Xiaoliang Luo, Guangzhi Sun, Martin Ferianc<br>Bradley C. Love
Building the experiment	Akilles Rechardt, Xiaoliang Luo, Bradley C. Love
Data analysis	Xiaoliang Luo, Akilles Rechardt, Bradley C. Love
Conceptualization and strategy	Xiaoliang Luo, Bradley C. Love
Figure creation	Xiaoliang Luo, Bati Yilmaz, Isil Bilgin<br>Chloe M. Hall, Guiomar Niso, Bradley C. Love
Useful input and suggestions on project	All authors
Commenting and editing on the manuscript	All authors

Table S.5: **Author contributions breakdown.** For each task, authors listed toward the beginning of the list contributed more.

Supplementary Figures

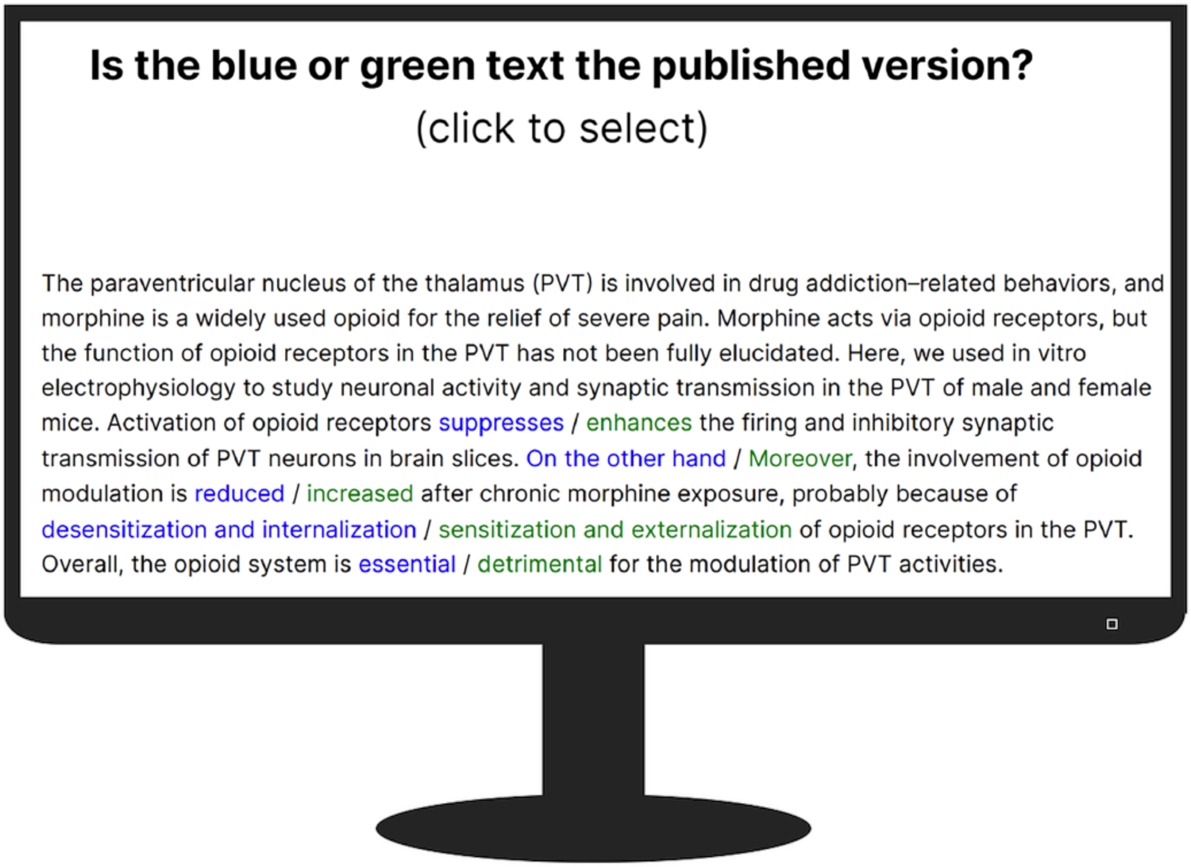

Figure S.1: **Study test interface**: Participants were instructed to select which version of the abstract was the original by clicking on either blue or green text to select that set of options. Various test cases may have varying numbers of alternatives, but a single click will choose all options of the same color.

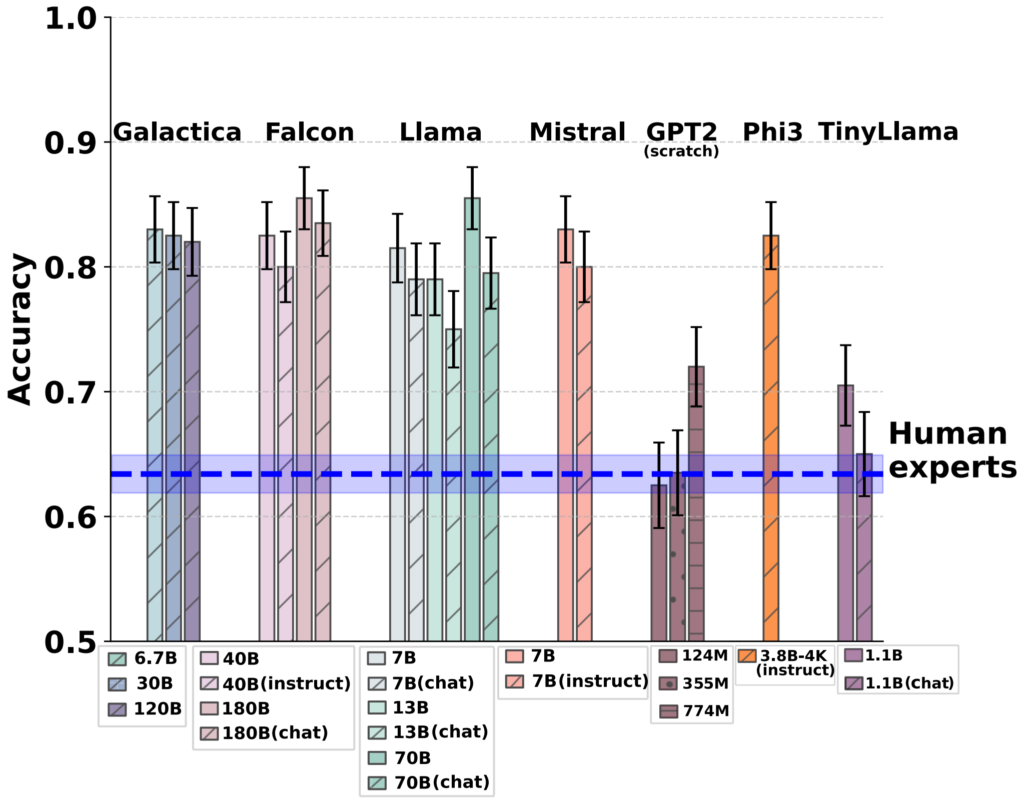

Figure S.2: **BrainBench performance across models of varying sizes and training data.** Models with 7 billion parameters or more achieve similar results on BrainBench, while their instruct/chat fine-tuned variants perform systematically worse. The GPT-2 series (124M, 355M, 774M) trained entirely on the neuroscience literature from scratch shows progressively better results, matching or surpassing human performance and closing the gap to larger models. Phi3, with half the size of the 7B models, achieves competitive results likely due to its high-quality training data. In contrast, TinyLlama, with 1.1 billion parameters, lags behind on BrainBench. Overall, performance is influenced by both model size and the quality and relevance of the training data. Error bars represent standard error of the accuracy. Each LLM was evaluated using 200 BrainBench test cases. In total, 171 human experts were evaluated with 1,011 trials covering the same BrainBench test cases.

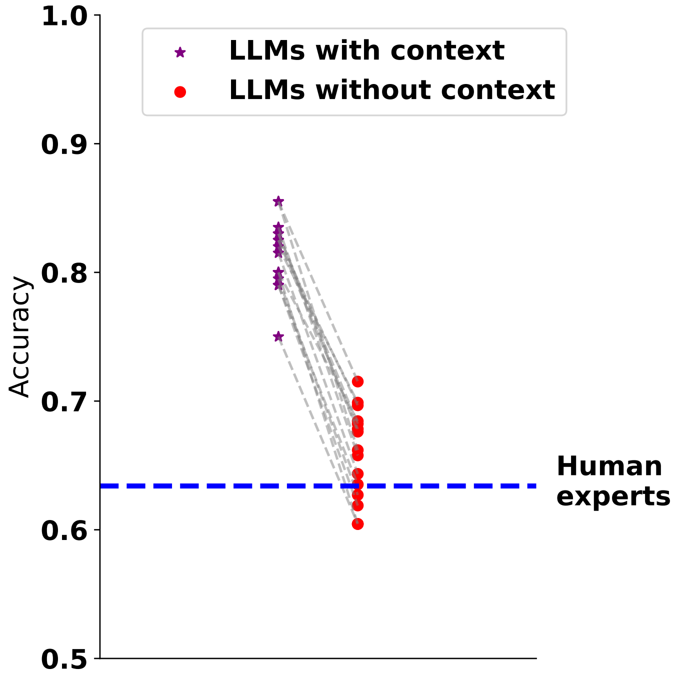

Figure S.3: **LLM integrates contextual information to succeed on BrainBench**. The removal of background and method sections from abstracts, with an evaluation based solely on individual sentences and result alternations, significantly impairs the performance of LLMs on BrainBench. LLMs' superior performance appears to arise from integrating information across the abstract.

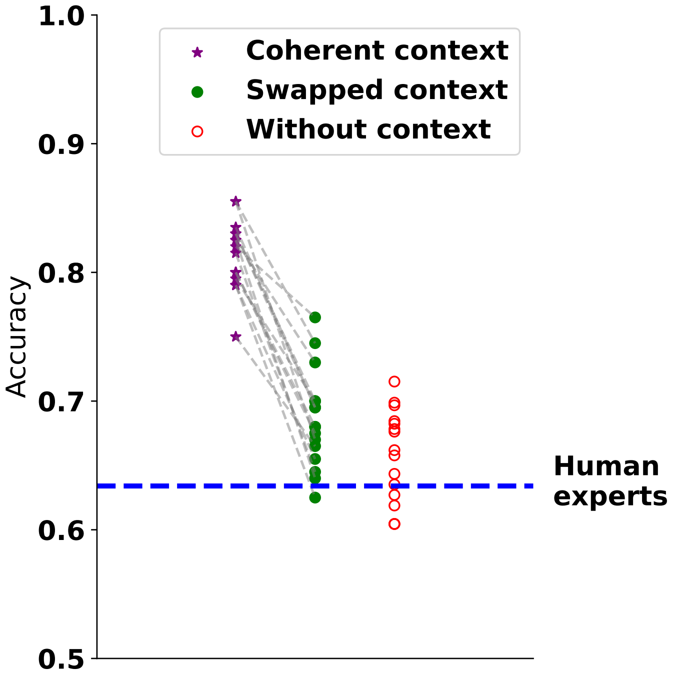

Figure S.4: **LLM performance with coherent, swapped and no context.** There is a significant performance decline when evaluating with coherent versus swapped contexts, with further degradation when context is absent.

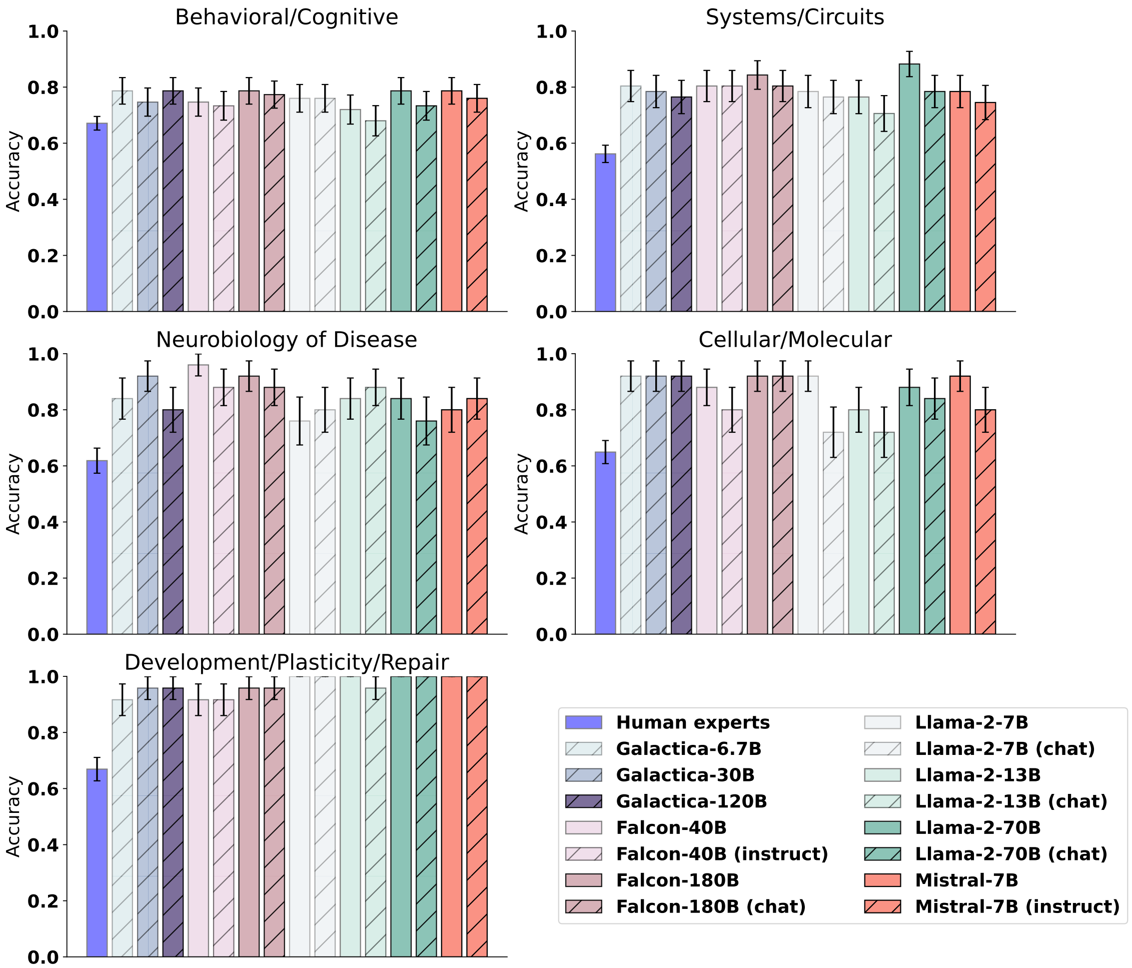

Figure S.5: **BrainBench performance by subfield.** Accuracy on BrainBench by subfields of neuroscience for human experts and LLMs. Error bars represent standard error of the accuracy. Breakdown of trials for each subfield: Behavioral/Cognitive ($n_{model} = 75, n_{human} = 374$), Systems/Circuits ($n_{model} = 51, n_{human} = 258$), Neurobiology of Disease ($n_{model} = 25, n_{human} = 118$), Cellular/Molecular ($n_{model} = 25, n_{human} = 134$), Development/Plasticity/Repair ($n_{model} = 24, n_{human} = 127$).

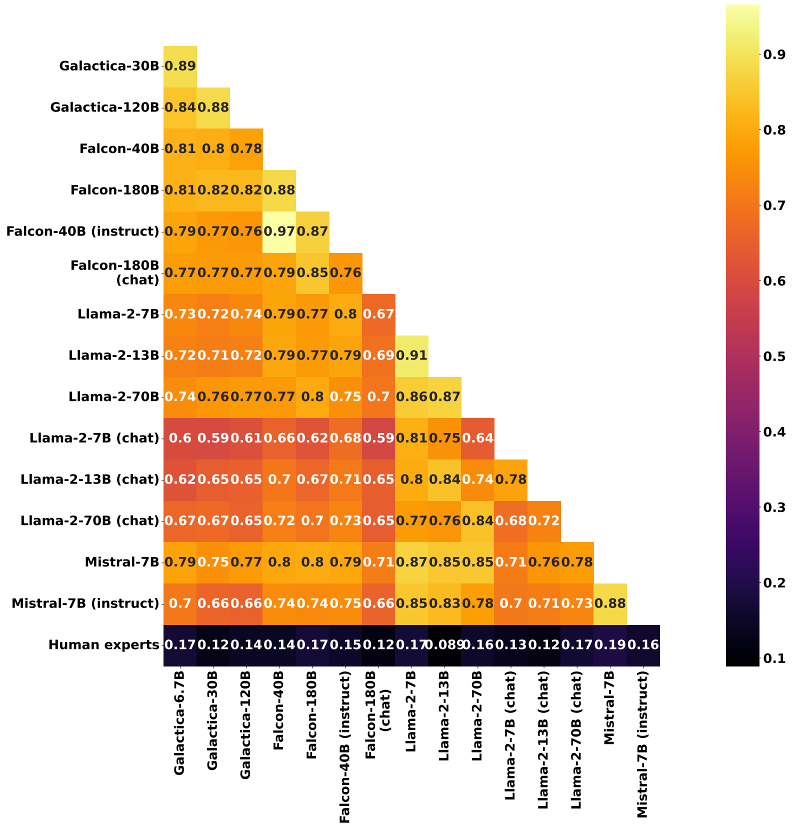

Figure S.6: **Item difficulty correlation among LLMs and human experts.** For LLMs, difference in perplexity between incorrect and correct abstracts is used to determine the relative difficulty of test cases. Mean accuracy is used for human experts. Spearman correlation is calculated for these difficulty measures. LLMs have an average correlation of 0.75 (± 0.08) whereas human experts have an average of 0.15 (± 0.03) with LLMs.

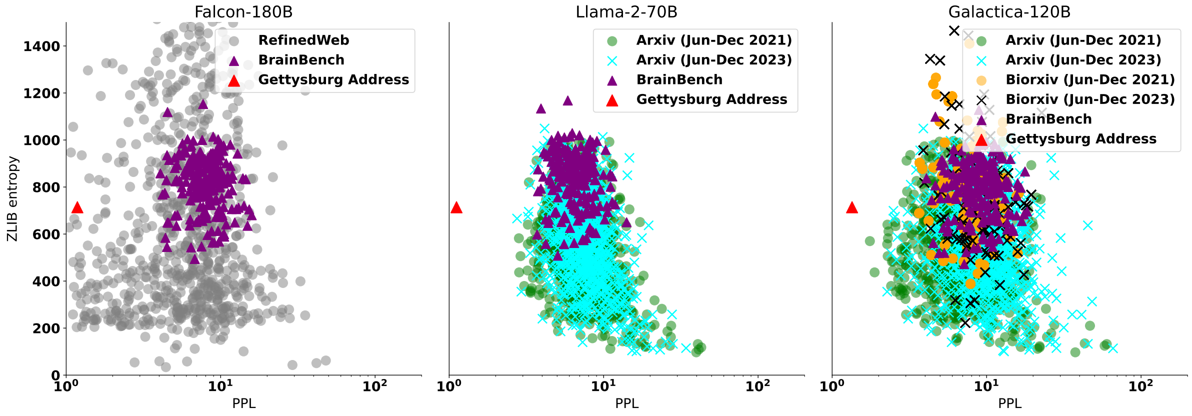

Figure S.7: **Memorization analysis.** Analyzing zlib entropy and perplexity ratio in text samples from known data sources, both included and excluded from LLMs training data, reveals no distinct signature for pre-trained data sources compared to those outside the training set. Although certain training data that occurs multiple times in the training set, like the Gettysburg Address, show signs of memorization, low-frequency training data show no sign of memorization. We analyzed the three largest LLMs we evaluated as they are most capable of memorizing data.

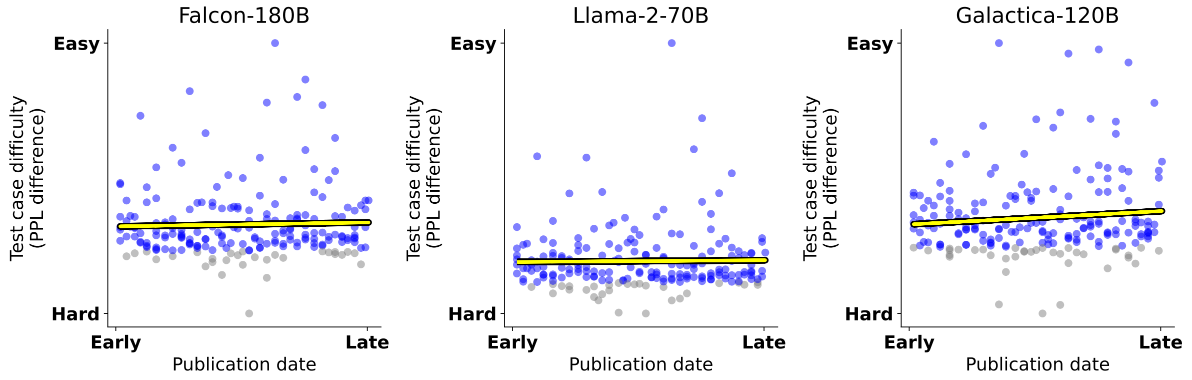

Figure S.8: **Spearman correlation between test cases' publication date and difficulty.** No correlation was found between the publication dates of BrainBench test cases and their difficulties in terms of perplexity differences to LLMs (Falcon-180B, $r(198) = 0.003$, $p = 0.972$; Llama-2-70B, $r(198) = -0.032$, $p = 0.652$; Galactica-120B, $r(198) = 0.033$, $p = 0.646$). Blue dots are test cases LLMs correctly classified; gray dots are test cases misclassified by LLMs. The significance of the correlation coefficients was determined by a t-Test with two-sided P values reported.

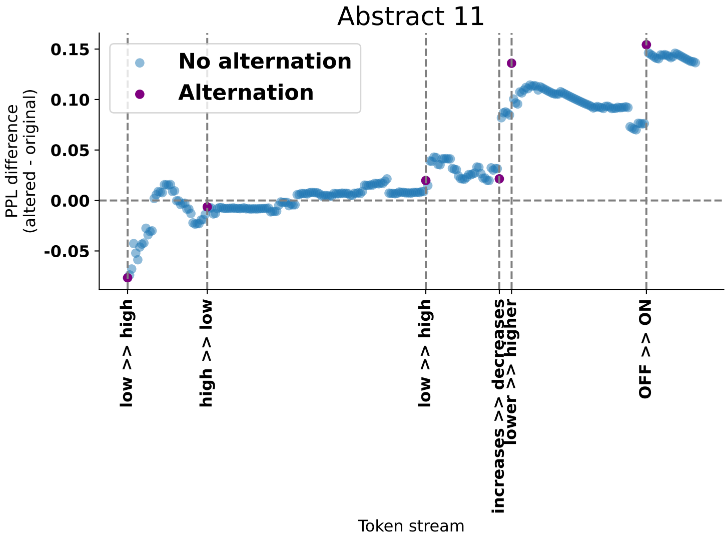

Figure S.9: **Perplexity difference at token level.** Red dots indicate alternations. For this example, the model makes an incorrect response at the first alternation. As the model processes more tokens, the perplexity difference increases between the altered and original leading to a correct response at the end (Mistral-7B-v0.1).

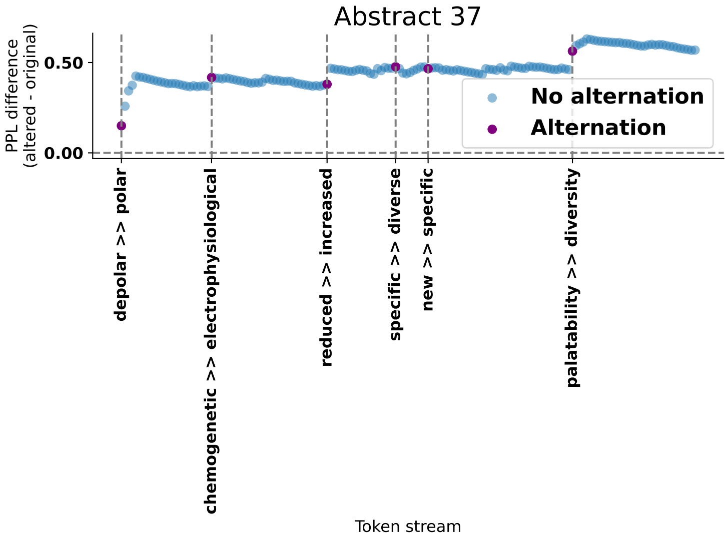

Figure S.10: **Perplexity difference at token level.** Red dots indicate alternations. For this example, the model makes a correct response starting from the first alternation already and stays being correct throughout the rest of the tokens (Mistral-7B-v0.1).

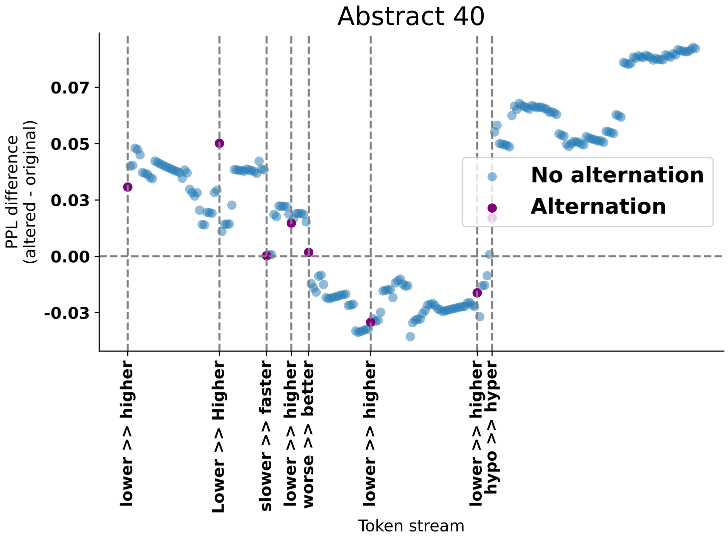

Figure S.11: **Perplexity difference at token level.** Red dots indicate alternations. For this example, the model makes a correct response starting from the first alternation. The model switches to an incorrect response after having processed about half of the abstract. The model's response flips once again at the final alternation and makes a correct response at the end (Mistral-7B-v0.1).

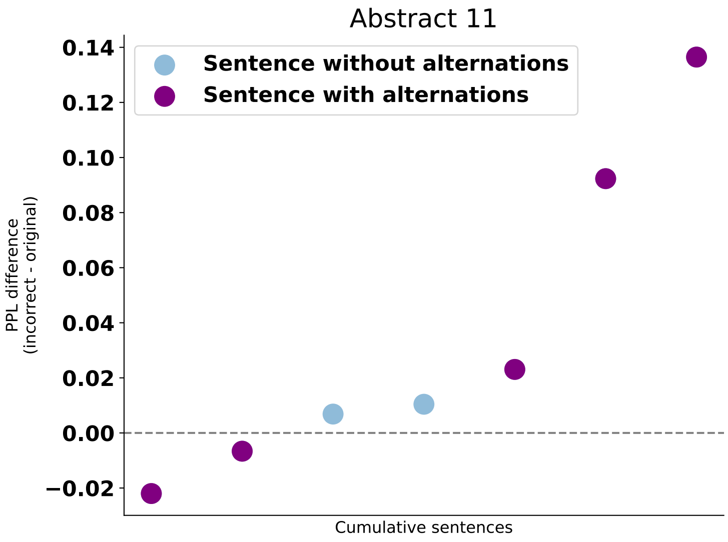

Figure S.12: **Perplexity difference at sentence level.** Red dots indicate the current sentence contains at least one alternations.

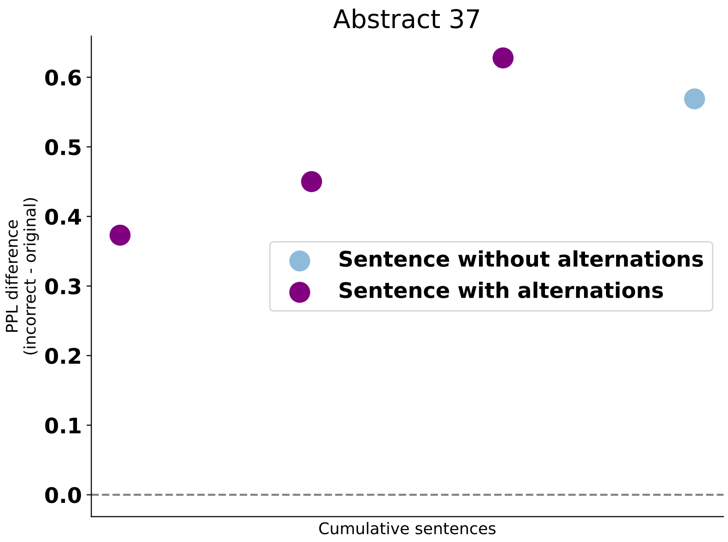

Figure S.13: **Perplexity difference at sentence level.** Red dots indicate the current sentence contains at least one alternations.

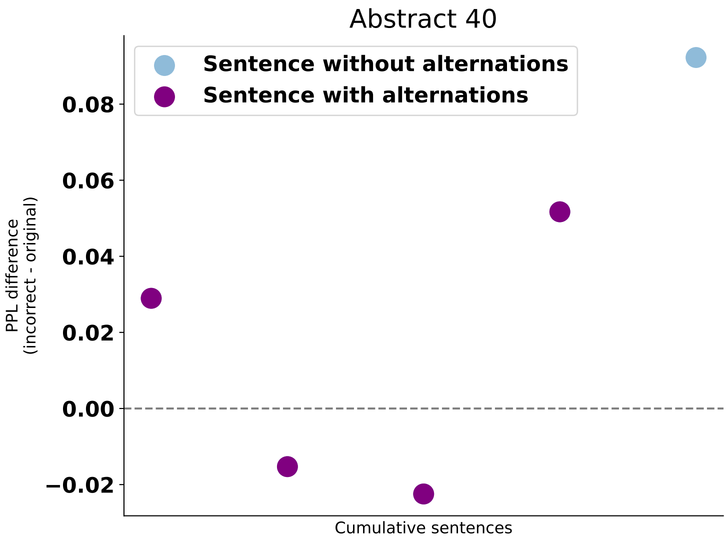

Figure S.14: **Perplexity difference at sentence level.** Red dots indicate the current sentence contains at least one alternations.

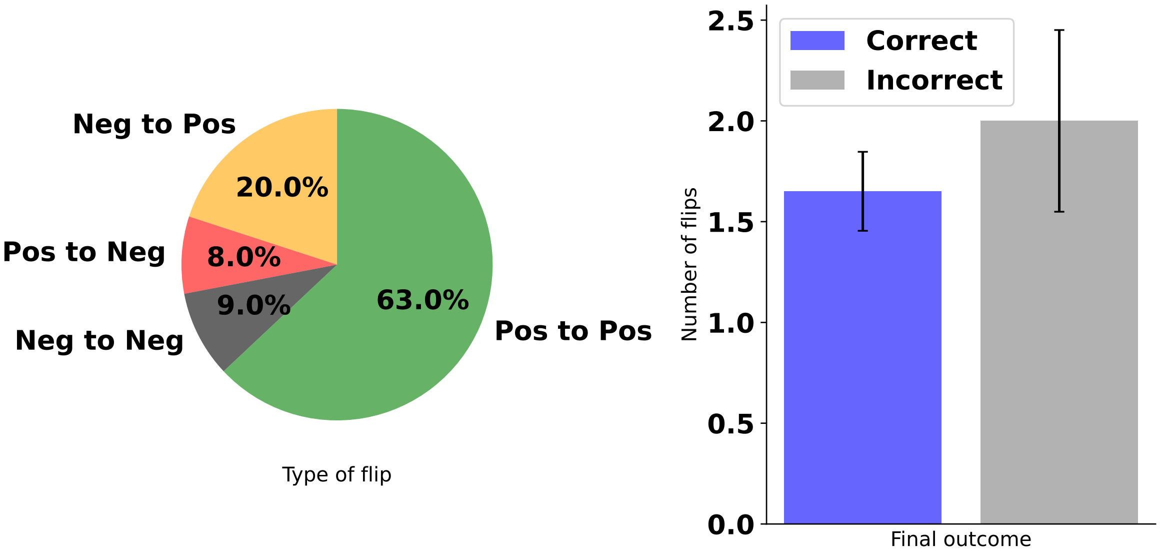

Figure S.15: **Cumulative perplexity differences at token level**. Left panel shows proportions of different decision flips of a model. A flip is determined by comparing the cumulative perplexity difference between the incorrect and the original abstracts until the first diverging token and the entire abstract. For example, a "Neg to Pos" flip suggests the model has a negative perplexity difference at the first diverging token and has a positive perplexity difference at the final token. Right panel shows the average number of perplexity difference sign flips in between tokens among abstracts classified correctly and incorrectly by the model. Error bars represent standard error of the accuracy ($n_{correct} = 164, n_{incorrect} = 34$).

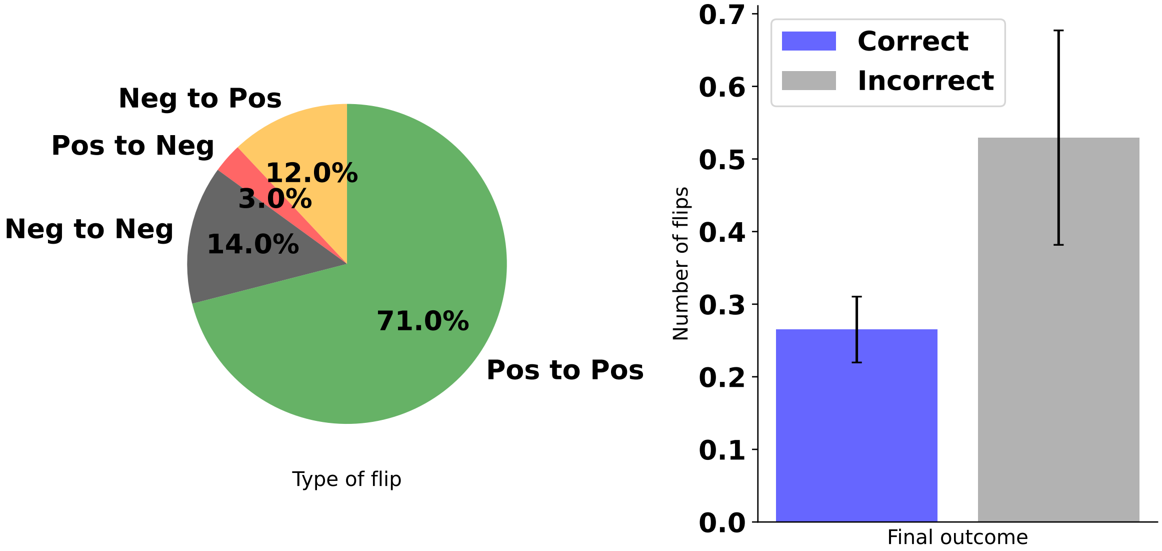

Figure S.16: **Cumulative perplexity differences at sentence level**. Left panel shows proportions of different decision flips of a model. A flip is determined by comparing the cumulative perplexity difference between the incorrect and the original abstracts until the sentence that contains the first diverging token and the entire abstract. For example, a “Neg to Pos” flip suggests the model has a negative perplexity difference at the sentence that contains the first diverging token and has a positive perplexity difference at the final token. Right panel shows the average number of perplexity difference sign flips in between sentences among abstracts classified correctly and incorrectly by the model. Error bars represent standard error of the accuracy ($n_{correct} = 164, n_{incorrect} = 34$).

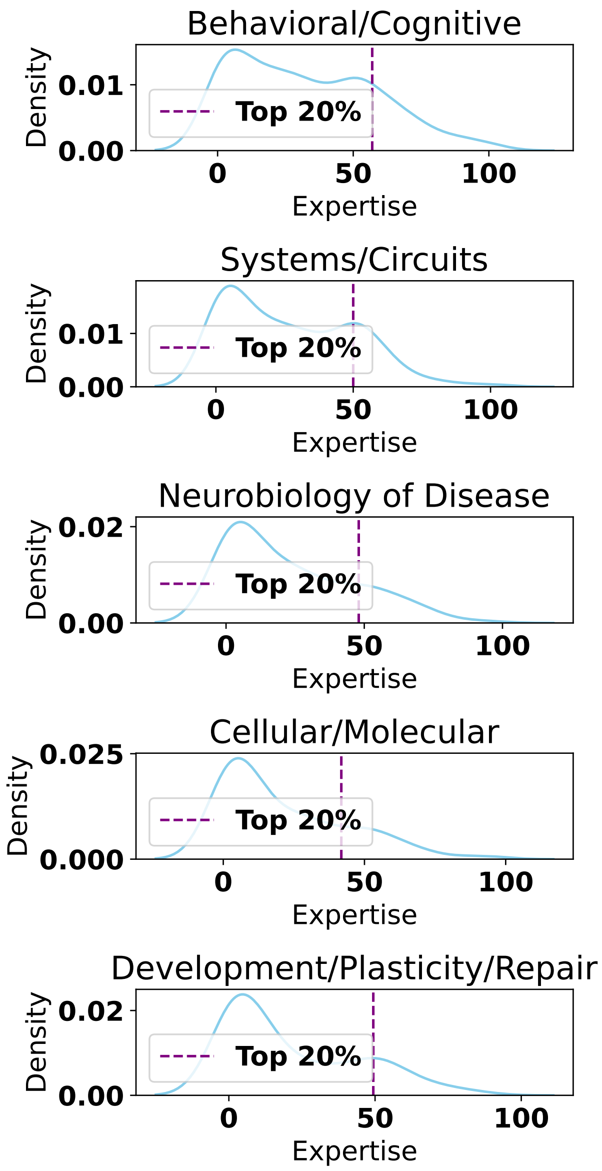

Figure S.17: **Expertise distribution across subfields.** The distribution of expertise are generally similar across subfields. The top 20% experts report expertise around or above 50/100.

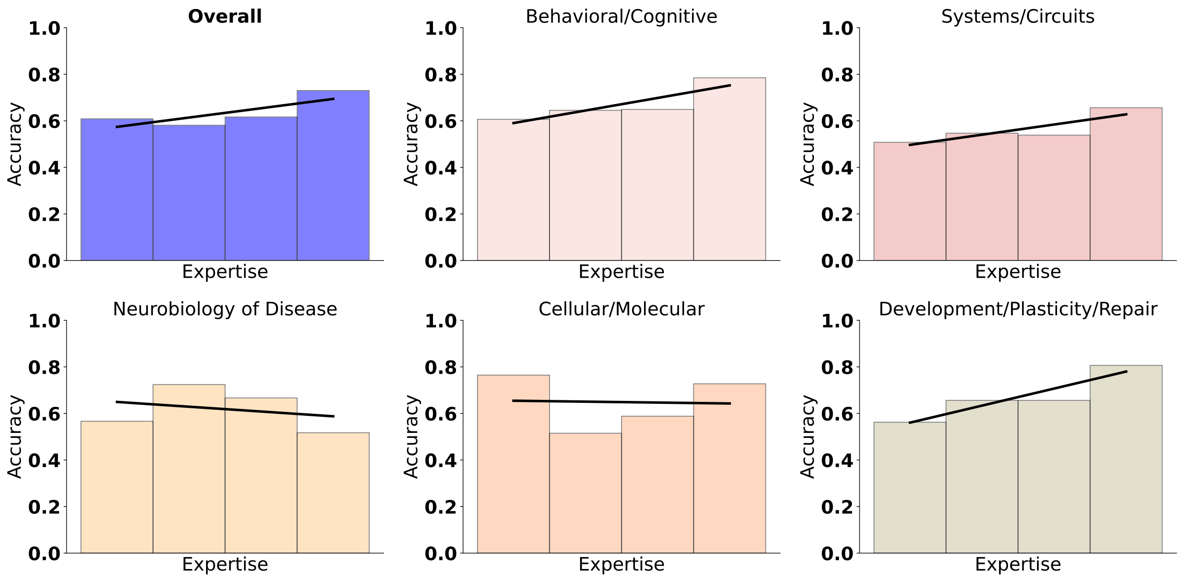

Figure S.18: **Expertise and BrainBench accuracies across subfields.** Self-reported expertise generally correlates positively with BrainBench accuracy. The pattern is not stronger and more uniform likely because expertise is on a per question basis as opposed to rated by entire subfield, individuals use the expertise scale differently, and expertise in general encompasses more than being good at prediction.

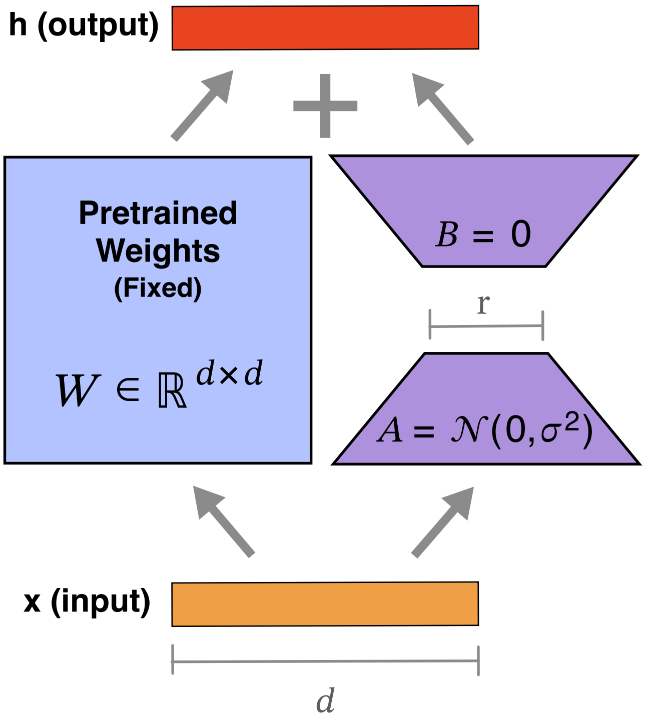

Figure S.19: **Low-Rank Adaptation (LoRA) for parameter-efficient large language model (LLM) fine-tuning.** Image adapted from[19]. Training LLMs from scratch can be computationally expensive, especially when the model has many billions of parameters and the training data are massive. Ideally, one would be able to take advantage of a previously trained LLM (i.e., a base model) and build from it. Toward this end, various Parameter Efficient Fine-Tuning (PEFT) techniques have been proposed. Rather than retrain the LLM, these techniques preserve the LLM's original pre-trained weights and train a small subset of additional parameters that either enhance an existing capability in the LLM or introduce a new one. In our case, we tune a base LLM to the neuroscience literature. This tuning strategy significantly alleviates the computational burden while achieving comparable performance to training a new LLM from scratch. Among many PEFT approaches, we adopted LoRA in our study to fine-tune the 7-Billion parameter version of the LLaMa2 model on neuroscience abstracts. LoRA fixes the pretrained model weights and injects trainable rank decomposition matrices into each layer of the transformer. During training, the same input sequence (or intermediate layer output) x is processed separately by the pretrained weights and the low-rank adaptation matrices (A and B). The low-rank matrices possess the only trainable parameters in the model. The final output h is computed as a coordinate-wise addition between the product of the pretrained weights and the adaptation matrices, which are further processed by subsequent layers.

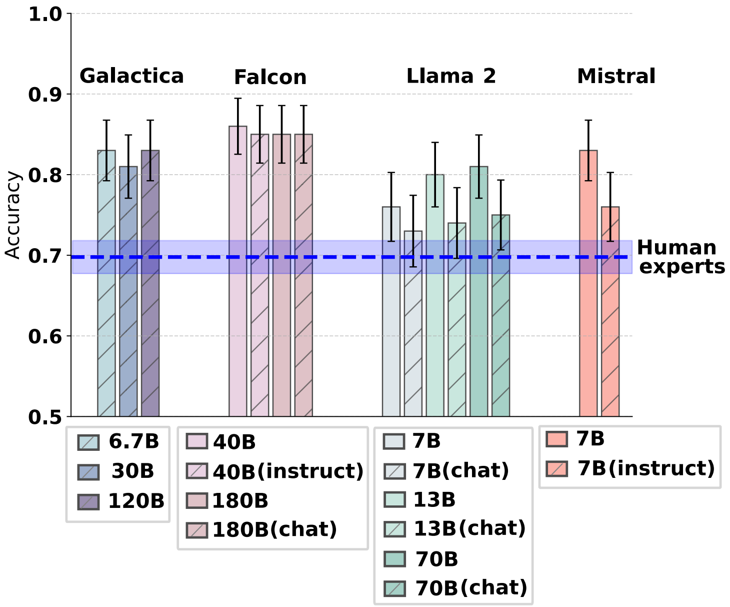

Figure S.20: **BrainBench performance of LLMs and human experts (using GPT-4 created test cases).** LLMs outperformed human experts on BrainBench ($t(14) = 9.20, p < 0.001$, cohen's $d = 3.36$, $95\% CI = 0.08 - 0.13$; two-sided). Base versions of models outperformed chat and instruct versions ($t(6) = 3.81, p = 0.008$, cohen's $d = 0.45$, $95\% CI = 0.01 - 0.06$; two-sided), which were tuned to be conversational with humans. Error bars represent standard error of the accuracy. Each model was evaluated over 100 BrainBench test cases. In total, 171 human experts were evaluated over the same test cases across 503 trials.

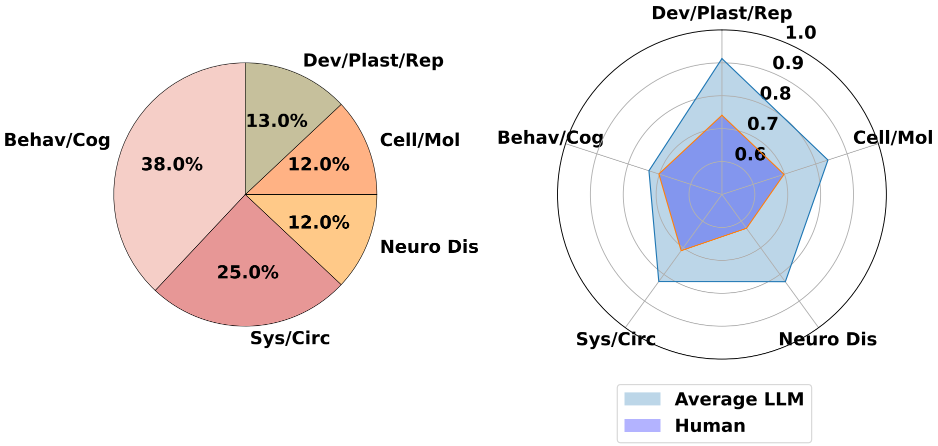

Figure S.21: **BrainBench performance breakdown by subfields of neuroscience (using GPT-4 created test cases).** The distribution of test cases across neuroscience subfields roughly mirrors the distribution of articles in the Journal of Neuroscience with Behavior/Cognitive over-represented. The mean performance of 15 LLMs and human experts is shown. LLMs outperformed human experts in all subfields.

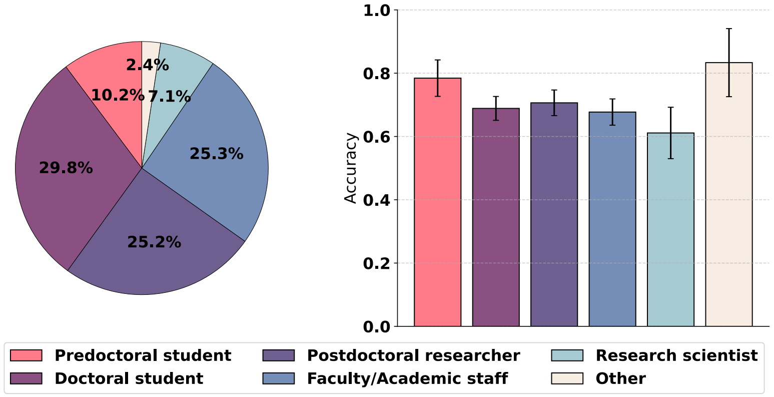

Figure S.22: **BrainBench performance of human experts belong to different self-reported career categories (using GPT-4 created test cases).** Participants were predoctoral students ($n_{trial} = 51$), doctoral students ($n_{trial} = 151$), postdoctoral researchers ($n_{trial} = 126$), faculty/academic staff ($n_{trial} = 127$) and research scientist ($n_{trial} = 36$) and others ($n_{trial} = 12$). Error bars represent standard error of the accuracy.

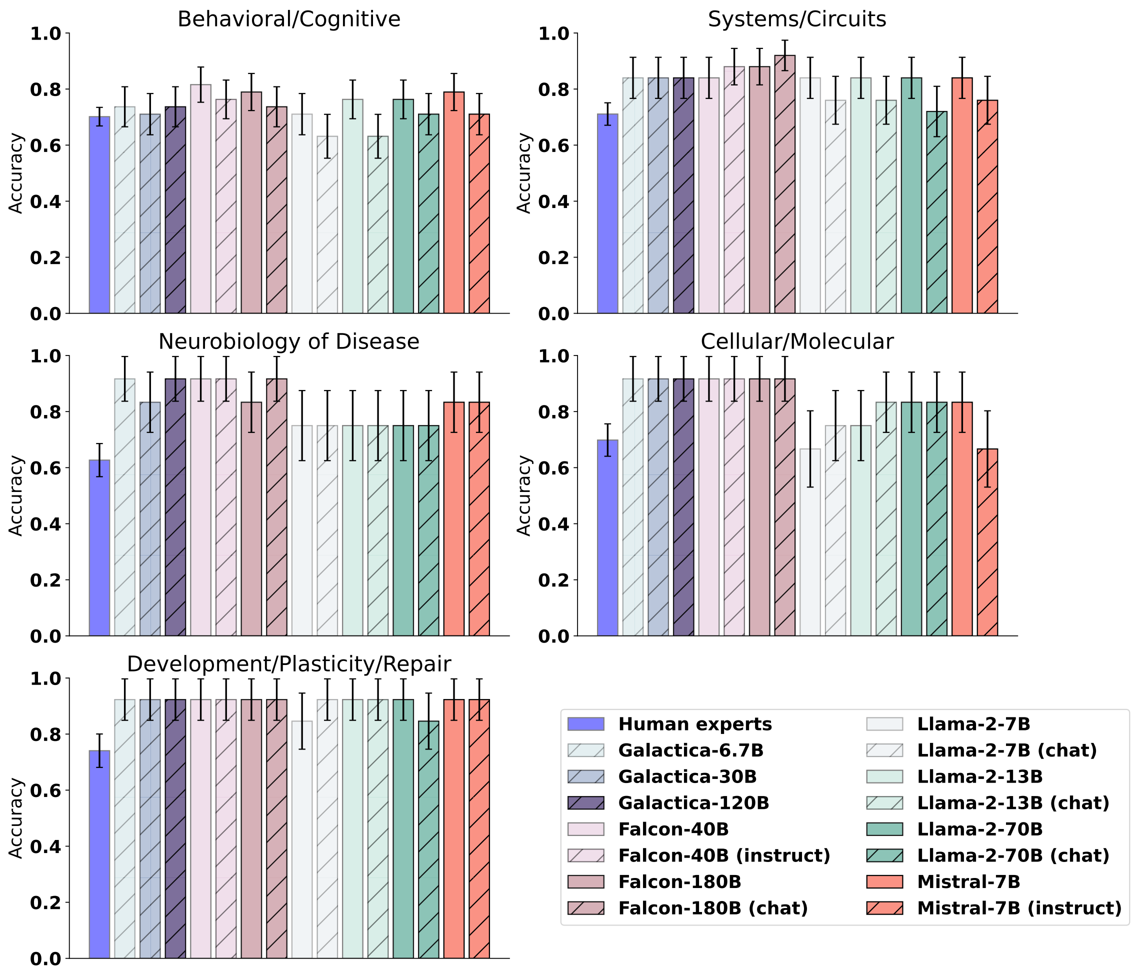

Figure S.23: **BrainBench performance breakdown (using GPT-4 created test cases).** Accuracy by subfields of neuroscience across human experts and LLMs. Error bars represent standard error of the accuracy. Breakdown of trials for each subfield: Behavioral/Cognitive ($n_{model} = 38, n_{human} = 191$), Systems/Circuits ($n_{model} = 25, n_{human} = 128$), Neurobiology of Disease ($n_{model} = 12, n_{human} = 67$), Cellular/Molecular ($n_{model} = 12, n_{human} = 63$), Development/Plasticity/Repair ($n_{model} = 13, n_{human} = 54$).

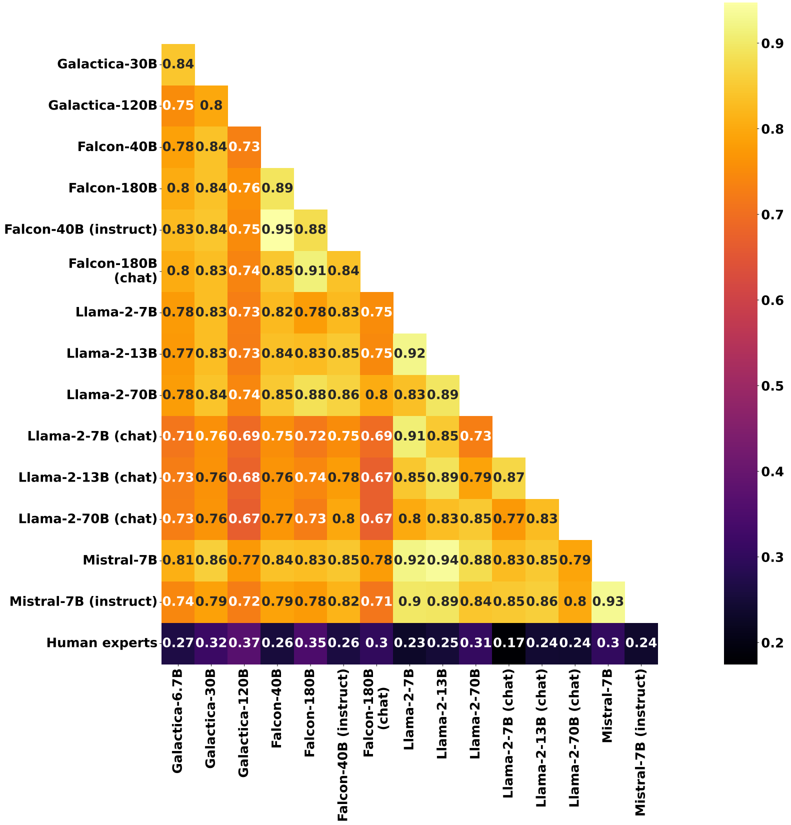

Figure S.24: **Test difficulty correlation among LLMs and human experts (using GPT-4 created test cases).** Difference in perplexity between incorrect and correct abstracts is used to determine the relative difficulty of test cases. Spearman correlation is computed between ranked difficulties by LLMs and human experts. LLMs have an average Spearman correlation of 0.83 (± 0.06) whereas human experts have an average of 0.27 (± 0.05) with LLMs.

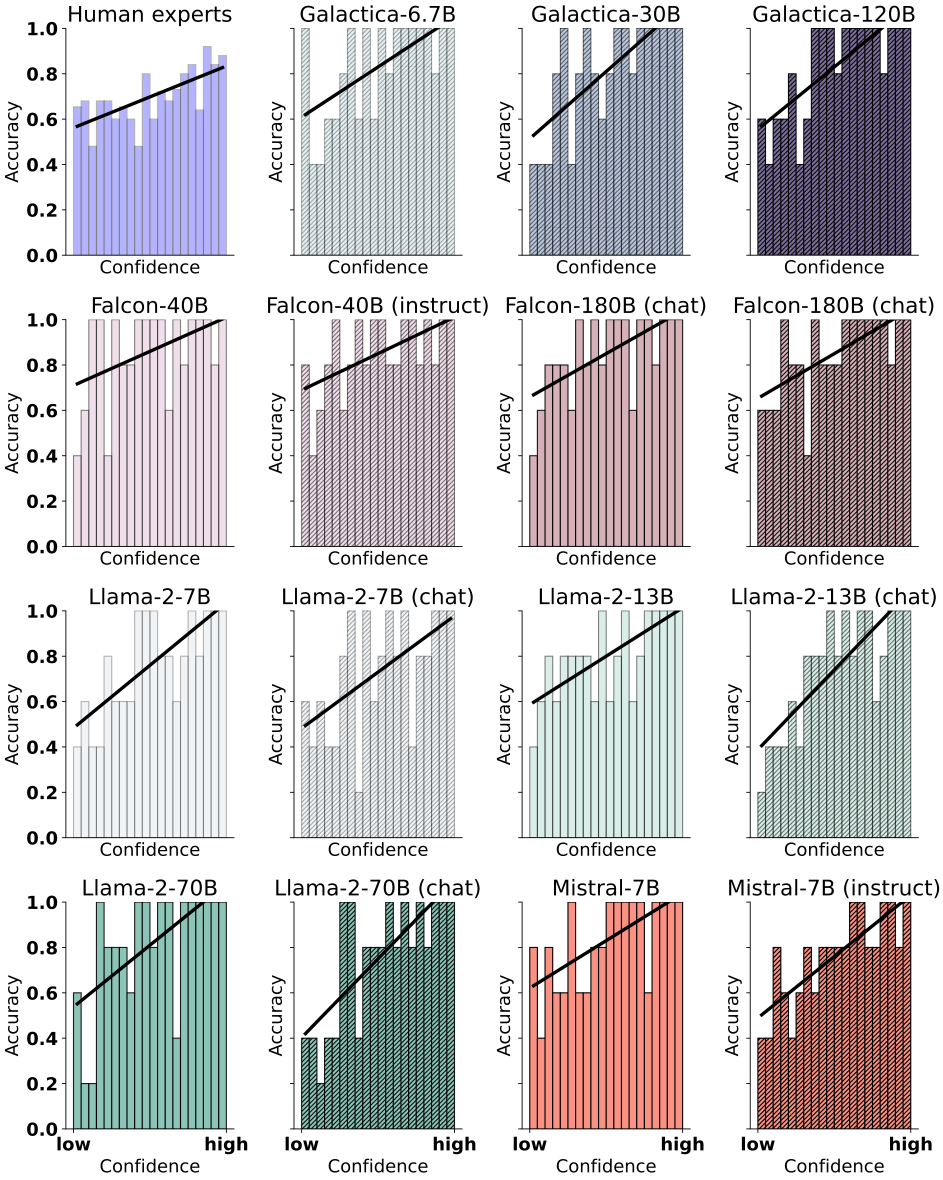

Figure S.25: **Accuracy and confidence are calibrated for human experts and LLMs (using GPT-4 created test cases).** When human experts and LLMs are confident in their BrainBench judgments, they are more likely to be correct. Confidence ratings were sorted and placed in equally-sized bins with the mean accuracy for items in that bin plotted. The black regression line's positive slope for human experts and all LLMs indicates that confidence is well calibrated (i.e., higher confidence corresponds to higher accuracy). Calibration is beneficial for human-machine teams.

Example #1

The amygdala plays a key role in the processing of itch and pain signals as well as emotion. A previous study [...] **Background**

[...] To test this possibility, prodynorphin (Pdyn)-Cre mice were used to optogenetically manipulate Pdyn+ CeA-to-PBN projections. **Method**

[...] We found that [...] Pdyn+ CeA-to-PBN projections [[inhibited, increased]] histamine-evoked and [...] Optogenetic stimulation of Pdyn+ CeA-to-PBN projections [[suppressed, enhanced]] the increase in Fos expression in the PBN. [...] **Result**

Example #2

Language comprehension requires the rapid retrieval and integration of contextually appropriate concepts ("semantic cognition"). Current neurobiological models [...] **Background**

[...] Through the use of fused functional magnetic resonance imaging and electroencephalography analysis in humans (n = 26 adults; 15 females), we elucidate a temporally and spatially specific neurobiological model [...] **Method**

[...] We find that [...] provide convergent evidence for the following progression: [[a hippocampal/anterior temporal, posterior temporal]] phonological semantic retrieval network [...] an inferior [[frontal, parietal]] semantic syntactic reappraisal network (~600 ms); and [...] **Result**

Figure S.26: **BrainBench examples.** For a test case, the background and the method sections of the abstract are kept unchanged. Alternative findings are introduced by human experts or GPT-4. Participants choose from two options, the original and the altered, with the aim of selecting the actual (i.e., original) finding. Blue shaded options are the original results. Green shaded options are the altered results.
