# Supplementary material for: Large language models surpass human experts in predicting neuroscience results
Source: Nat Hum Behav. 2024 Nov 27;9(2):305–15. doi: 10.1038/s41562-024-02046-9 (PMC11860209; doi:10.1038/s41562-024-02046-9)
Supplement: Supplementary file 2 — Reporting Summary [file 41562_2024_2046_MOESM2_ESM.pdf]

nature portfolio

Corresponding author(s): Xiaoliang Luo

Last updated by author(s): Sep 25, 2024

Reporting Summary

Nature Portfolio wishes to improve the reproducibility of the work that we publish. This form provides structure for consistency and transparency in reporting. For further information on Nature Portfolio policies, see our Editorial Policies and the Editorial Policy Checklist.

Statistics

For all statistical analyses, confirm that the following items are present in the figure legend, table legend, main text, or Methods section.

n/a	Confirmed	
☐	☒	The exact sample size (*n*) for each experimental group/condition, given as a discrete number and unit of measurement
☒	☐	A statement on whether measurements were taken from distinct samples or whether the same sample was measured repeatedly
☐	☒	The statistical test(s) used AND whether they are one- or two-sided<br>*Only common tests should be described solely by name; describe more complex techniques in the Methods section.*
☒	☐	A description of all covariates tested
☒	☐	A description of any assumptions or corrections, such as tests of normality and adjustment for multiple comparisons
☐	☒	A full description of the statistical parameters including central tendency (e.g. means) or other basic estimates (e.g. regression coefficient) AND variation (e.g. standard deviation) or associated estimates of uncertainty (e.g. confidence intervals)
☐	☒	For null hypothesis testing, the test statistic (e.g. *F*, *t*, *r*) with confidence intervals, effect sizes, degrees of freedom and *P* value noted<br>*Give P values as exact values whenever suitable.*
☒	☐	For Bayesian analysis, information on the choice of priors and Markov chain Monte Carlo settings
☒	☐	For hierarchical and complex designs, identification of the appropriate level for tests and full reporting of outcomes
☐	☒	Estimates of effect sizes (e.g. Cohen's *d*, Pearson's *r*), indicating how they were calculated

Our web collection on statistics for biologists contains articles on many of the points above.

Software and code

Policy information about availability of computer code

Data collection

Human experts data was collected online using the Gorilla platform (Build-2023-12-04). Data produced by GPT-4 was created via Azure OpenAI API; version 2023-05-15.

Data analysis

Software packages used for data analysis:

# Name	Version	Build	Channel
accelerate	0.24.1	pypi_0	pypi
bitsandbytes	0.37.0	pypi_0	pypi
cudatoolkit	11.3.1	h2bc3f7f_2	
datasets	2.15.0	pypi_0	pypi
deepspeed	0.12.6	pypi_0	pypi
matplotlib	3.7.0	pypi_0	pypi
nltk	3.8.1	pypi_0	pypi
numpy	1.24.3	py39hf6e8229_1	
openai	1.3.5	pypi_0	pypi
pandas	2.0.1	pypi_0	pypi
peft	0.3.0	pypi_0	pypi
pubget	0.0.8	pypi_0	pypi
python	3.9.16	h7a1cb2a_2	
scikit-learn	1.2.2	pypi_0	pypi
scipy	1.10.1	pypi_0	pypi
seaborn	0.13.1	pypi_0	pypi

torch 2.0.1 pypi_0 pypi
transformers 4.36.0 pypi_0 pypi
wandb 0.15.2 pypi_0 pypi
xformers 0.0.22 pypi_0 pypi

Algorithms (LLMs) used:
Galactica-6.7B
Galactica-30B
Galactica-120B
Falcon-40B
Falcon-40B (instruct)
Falcon-180B
Falcon-180B (chat)
Llama-2-7B
Llama-2-7B (chat)
Llama-2-13B
Llama-2-13B (chat)
Llama-2-70B
Llama-2-70B (chat)
Mistral-7B
Mistral-7B (instruct)
GPT2-124M
GPT2-335M
GPT2-774M
Phi3-3.8B-4K (instruct)
TinyLlama-1.1B-v1.1
TinyLlama-1.1B-Chat-v1.0

For manuscripts utilizing custom algorithms or software that are central to the research but not yet described in published literature, software must be made available to editors and reviewers. We strongly encourage code deposition in a community repository (e.g. GitHub). See the Nature Portfolio guidelines for submitting code & software for further information.

Data

Policy information about availability of data

All manuscripts must include a data availability statement. This statement should provide the following information, where applicable:

- Accession codes, unique identifiers, or web links for publicly available datasets
- A description of any restrictions on data availability
- For clinical datasets or third party data, please ensure that the statement adheres to our policy

Human participant data, and intermediate data generated via simulations and analyses are publicly available at https://github.com/braingpt-lovelab/BrainBench
Model weights and training data are available at https://huggingface.co/BrainGPT

Raw training data are sourced from PubMed and PubMed Central Open Access Subset (PMC OAS) using the Entrez Programming Utilities (E-utilities) API (https://www.ncbi.nlm.nih.gov/books/NBK25497/) and the pubget Python package (https://pypi.org/project/pubget/), respectively.

Research involving human participants, their data, or biological material

Policy information about studies with human participants or human data. See also policy information about sex, gender (identity/presentation), and sexual orientation and race, ethnicity and racism.

Reporting on sex and gender	Data on sex or gender was not relevant for the purpose of the current study which focuses on participants' expertise in Neuroscience.
Reporting on race, ethnicity, or other socially relevant groupings	No such data was collected in the study
Population characteristics	Participant population includes 171 participants consisted of 51 doctoral students, 43 faculty/academic staff, 43 post-doctoral researchers, 18 predoctoral students, 12 research scientists, and 4 classified as “other”. Participants’ mean experience in Neuroscience was 10.1 years
Recruitment	We recruited 202 neuroscience experts via social media and an email newsletter. We excluded 31 participants for failing to answer both catch trials correctly, not providing confidence or expertise ratings during the entire experiment, and self-reported cheating. To our knowledge, there were no sources of self-selection bias that would be likely to impact the study findings as a result of this recruitment procedure.
Ethics oversight	Experimental Psychology Ethics Board, UCL

Note that full information on the approval of the study protocol must also be provided in the manuscript.

Field-specific reporting

Please select the one below that is the best fit for your research. If you are not sure, read the appropriate sections before making your selection.

☐ Life sciences ☒ Behavioural & social sciences ☐ Ecological, evolutionary & environmental sciences

For a reference copy of the document with all sections, see nature.com/documents/nr-reporting-summary-flat.pdf

Behavioural & social sciences study design

All studies must disclose on these points even when the disclosure is negative.

Study description	The data consist of numerical responses to test items from the BrainBench dataset. Data reported in the manuscript are quantitative numeric scores assigned to each test item. The design follows a between-samples comparison of Large Language Models (LLMs) against human experts.
Research sample	LLMs: Galactica-6.7B, Galactica-30B, Galactica-120B, Falcon-40B, Falcon-40B (instruct), Falcon-180B, Falcon-180B (chat), Llama-2-7B, Llama-2-7B (chat), Llama-2-13B, Llama-2-13B (chat), Llama-2-70B, Llama-2-70B (chat), Mistral-7B, Mistral-7B (instruct), GPT2-124M, GPT2-335M, GPT2-774M, Phi3-3.8B-4K (instruct), TinyLlama-1.1B-v1.1, TinyLlama-1.1B-Chat-v1.0. We chose a wide range of models of varying sizes and trained by different organizations developed at different times on diverse data. Human experts: 171 participants consisted of 51 doctoral students, 43 faculty/academic staff, 43 post-doctoral researchers, 18 predoctoral students, 12 research scientists, and 4 classified as “other”. Participants’ mean experience in Neuroscience was 10.1 years. We did not specify particular demographics because the main comparison of interest was human experts vs. LLM performance on predicting Neuroscience results and we had no reason to build a priori hypotheses about demographics. Recruitment was not restricted to any country. We opted for the most diverse yet representative and compute-affordable models and recruited as many qualified neuroscientists as possible given the resources available to us at the time of the study.
Sampling strategy	LLMs: sampled most representative models across sizes, development time and training data. Human experts: convenience sampling subject to pre-established exclusion criteria through the Prolific platform.
Data collection	First, participants were briefed on the experimental task and provided their informed consent to proceed to the experiment. Demographic information was then collected, including gender identity, age, country, current position, and years of experience in Neuroscience research, broadly construed. Next, participants completed a practice trial using the same testing format as the actual test cases. This trial was used to familiarize participants with the format of the task with the screen proceeding only once participants had made the correct choice based on common sense. Following this, 9 test trials and 2 catch trials commenced, where participants selected one version of each trial abstract. Out of the 9 test trials, 6 were randomly sampled human-created test cases and 3 were randomly sampled from the pool of machine-created items. We ensured that each test case is sampled approximately an equal number of times across all participants. To achieve this, we maintained a global counter that keeps track of how frequently each test case has been used. As a result, the next participant's sample will always be drawn from those test cases that have been used less frequently. Notably, the number of alterations varies between test cases, but the design allowed a single click to automatically select between the two abstract options. Participants made one decision per test case, regardless of the number of alternations.<br><br>Subsequently, participants were required to rate their confidence and expertise using slider bars. The confidence slider had a range from ``lower" on the left to ``higher" on the right, while the expertise slider spanned from “not at all” on the left to “very much so” on the right, both internally implementing a 1-100 scaling. Additionally, participants indicated whether they had encountered the study previously before proceeding to the next trial. Upon completing the 11 trials, participants were debriefed on which trials they got correct and were subsequently asked to indicate whether they engaged in any form of cheating during the study. We hosted the study entirely on the Gorilla platform.
Timing	11/12/2023 - 22/12/2023
Data exclusions	We apply several pre-established exclusion criteria. First, individuals who failed to answer both catch trials correctly were not included in the data analyses. Second, participants who did not make adjustments to the sliders (i.e., expertise and confidence) during any of the trials were excluded. Additionally, trials where participants recognized the abstract content were omitted from the analysis. Furthermore, trials with reaction times less than 5 seconds were excluded. Lastly, participants who admitted to using external resources or engaging in cheating behaviors, as indicated by a checkbox in the debriefing form, were not considered in the final data analysis. Exactly 31 participants were excluded.
Non-participation	795 people did not finish the entire test suite.
Randomization	Participants were not assigned to experimental groups. They were presented randomly with BrainBench test cases on the Gorilla platform as detailed in Data collection.

Reporting for specific materials, systems and methods

We require information from authors about some types of materials, experimental systems and methods used in many studies. Here, indicate whether each material, system or method listed is relevant to your study. If you are not sure if a list item applies to your research, read the appropriate section before selecting a response.

Materials & experimental systems

n/a	Involved in the study
☒	☐ Antibodies
☒	☐ Eukaryotic cell lines
☒	☐ Palaeontology and archaeology
☒	☐ Animals and other organisms
☒	☐ Clinical data
☒	☐ Dual use research of concern
☒	☐ Plants

Methods

n/a	Involved in the study
☒	☐ ChIP-seq
☒	☐ Flow cytometry
☒	☐ MRI-based neuroimaging

Plants

Seed stocks	*Report on the source of all seed stocks or other plant material used. If applicable, state the seed stock centre and catalogue number. If plant specimens were collected from the field, describe the collection location, date and sampling procedures.*
Novel plant genotypes	*Describe the methods by which all novel plant genotypes were produced. This includes those generated by transgenic approaches, gene editing, chemical/radiation-based mutagenesis and hybridization. For transgenic lines, describe the transformation method, the number of independent lines analyzed and the generation upon which experiments were performed. For gene-edited lines, describe the editor used, the endogenous sequence targeted for editing, the targeting guide RNA sequence (if applicable) and how the editor was applied.*
Authentication	*Describe any authentication procedures for each seed stock used or novel genotype generated. Describe any experiments used to assess the effect of a mutation and, where applicable, how potential secondary effects (e.g. second site T-DNA insertions, mosiacism, off-target gene editing) were examined.*
